# Combined quantum tunnelling and dielectrophoretic trapping for molecular analysis at ultra-low analyte concentrations

**DOI:** 10.1038/s41467-021-21101-x

**Published:** 2021-02-10

**Authors:** Longhua Tang, Binoy Paulose Nadappuram, Paolo Cadinu, Zhiyu Zhao, Liang Xue, Long Yi, Ren Ren, Jiangwei Wang, Aleksandar P. Ivanov, Joshua B. Edel

**Affiliations:** 1 State Key Laboratory of Modern Optical Instrumentation, College of Optical Science and Engineering; International Research Center for Advanced Photonics, Zhejiang University, Hangzhou, China; 2 Department of Chemistry, Molecular Science Research Hub, Imperial College London, London, UK; 3 Innovation Institute for Artificial Intelligence in Medicine, Zhejiang University, Hangzhou, China; 4 Center of Electron Microscopy and State Key Laboratory of Silicon Materials, School of Materials Science and Engineering, Zhejiang University, Hangzhou, China

**Keywords:** Analytical chemistry, Nanoscale devices, Techniques and instrumentation

## Abstract

Quantum tunnelling offers a unique opportunity to study nanoscale objects with atomic resolution using electrical readout. However, practical implementation is impeded by the lack of simple, stable probes, that are required for successful operation. Existing platforms offer low throughput and operate in a limited range of analyte concentrations, as there is no active control to transport molecules to the sensor. We report on a standalone tunnelling probe based on double-barrelled capillary nanoelectrodes that do not require a conductive substrate to operate unlike other techniques, such as scanning tunnelling microscopy. These probes can be used to efficiently operate in solution environments and detect single molecules, including mononucleotides, oligonucleotides, and proteins. The probes are simple to fabricate, exhibit remarkable stability, and can be combined with dielectrophoretic trapping, enabling active analyte transport to the tunnelling sensor. The latter allows for up to 5-orders of magnitude increase in event detection rates and sub-femtomolar sensitivity.

## Abstract (web summary)

Probes that effectively utilize quantum tunneling are sought after for high-resolution study of nanoscale objects. Here the authors present an easily fabricated probe of two nanoelectrodes that enables highly sensitive quantum-tunneling-based sensing of single molecules.

## Introduction

The ability to perform accurate identification and detection of individual molecules in solution is invaluable in many applications. This is especially true when dealing with small sample volumes and low analyte concentrations. More generally, most experimental observations of physical and biological systems provide a measurement of ensemble averages. In contrast, single-molecule methods allow for the observation of dynamics and kinetics of individual populations which is exceptionally valuable when trying to better understand low abundant species. Over the past few decades, several techniques, including a range of electrical and optical methods, with sufficient sensitivity have been developed that allow probing of single molecules^1–7^. Among these methods, quantum mechanical tunnelling (QMT)-based sensors are particularly attractive due to their remarkable spatial resolution, high sensitivity and potential for integration into larger-scale platforms^2,4,8,9^.

At the heart of these sensors, is the transport of electrons across sub-5 nm electrode gaps or junctions that relies on the QMT effect. When a voltage is applied to the electrodes, electrons tunnel across the gap producing a tunnelling current with a magnitude that is dependent on the applied voltage, the width of the gap and the medium in the gap^10^. Furthermore, the inclusion of a molecule in the tunnelling junction alters the current, giving rise to characteristic signals, whose amplitude and duration can reveal the nature and properties of the analyte. It has been shown both theoretically and experimentally that the tunnelling current provides exceptional spatial resolution down to atomic scales and can even be used to distinguish between individual nucleotides^2,11,12^. This capability holds much promise as a viable route towards achieving future generation nucleic acid sequencing and potentially even protein sequencing^2,3,12–17^.

At present, QMT detection typically requires complex fabrication and instrumentation. For example, the most widely used configuration is based on a scanning tunnelling microscope (STM), where piezo controllers and tunnelling current amplifiers/controllers are used to form a gap between an atomically sharp tunnelling probe and a flat conductive surface^14,15,18,19^. Alternatively, functional tunnelling junctions can be achieved using a variety of techniques such as break-junction^12,16,17^, electromigration^20^, electrodeposition^21,22^, or directly using advanced lithographic techniques to fabricate planar or stacked devices^13,23–25^. There is currently much to improve in terms of simplicity of fabrication and the possibility to operate in a standalone configuration, using conventional measurement instrumentation. Furthermore, detection largely relies on transport of the analytes by diffusion into the tunnelling junction and there is limited or no control over molecular transport^26,27^. This is compounded by the fact that confinement of an analyte in a tunnelling junction is entropically unfavourable, leading to low detection event throughput and the requirement for high analyte concentrations. Ultimately these challenges are, to a large extent, the reason why QMT sensors are being held back for broader analytical implementation.

**Fig. 1:**
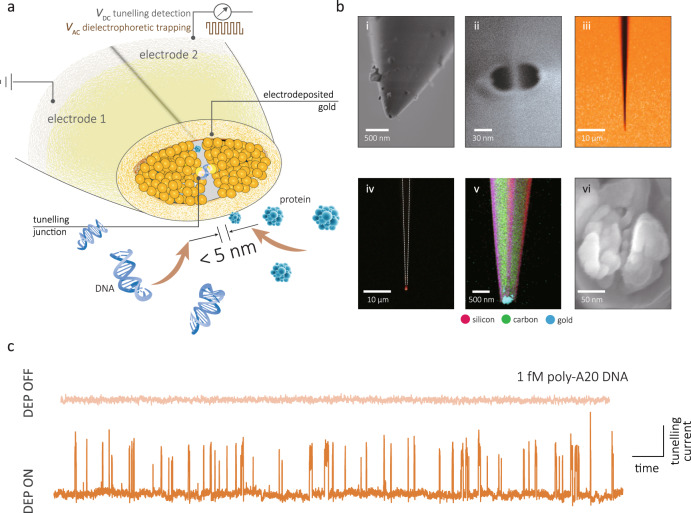
Dielectrophoretic trapping-combined quantum mechanical tunnelling sensors for the detection of DNA and proteins. **a** Conceptual design of the QMT probe. DEP trapping was initiated by applying an AC electric field while tunnelling was performed under a constant DC bias. Single-molecule tunnelling detection was performed on mononucleotides, small DNA oligomers and proteins. **b** The tunnelling junction was made from (i) double-barrelled glass capillaries that were laser pulled to nanoscale dimensions, as shown in (ii). Nanoscale electrodes were made using a combination of pyrolytic deposition of butane and electrodeposition of gold with real-time feedback. Optical images (iii–iv), scanning TEM-energy dispersive spectrometry (v) and SEM imaging (vi) clearly show gold at the tip of the probe. **c** Typical tunnelling current–time trace as shown for DNA at a concentration of 1 fM with and without applying a dielectrophoretic trap. The scale bar along the *x-* axis is 5 ms and along the *y-*axis is 20 nA.

We address the above limitations by reporting on a QMT probe that is based on a simple yet robust fabrication protocol that consists of individually addressable nanoelectrodes in a standalone double-barrel nanoprobe. Importantly, we show that the QMT electrodes are compatible and can be used in combination with dielectrophoretic (DEP) trapping to capture molecules subjected to a non-uniform electric field^28–30^, and enable the active pre-concentration of the analytes for high throughput analysis or detection of single molecules in highly dilute environments (Fig. 1).

## Results and discussion

### Fabrication of the QMT nanoprobes

The fabrication and characterisation steps for making the QMT probes, including a flow chart, can be found in Supplementary Section 1 and Supplementary Fig. 1. Briefly, the probe was fabricated from theta-shaped dual-barrel quartz capillary, which was laser pulled to a sharp tip terminating in two closely spaced nanopores (25 ± 12 nm in diameter) separated by a quartz septum (15 ± 5 nm in width). Both barrels were filled with carbon by pyrolysis, resulting in two semi-elliptical coplanar carbon nanoelectrodes, at the tip of the capillary (Fig. 1b and Supplementary Fig. 2)^30,31^. Elemental analysis of the tip confirmed that the pyrolytic carbon was fully deposited inside the quartz capillary (Supplementary Fig. 2e and Supplementary Table 1).

**Fig. 2:**
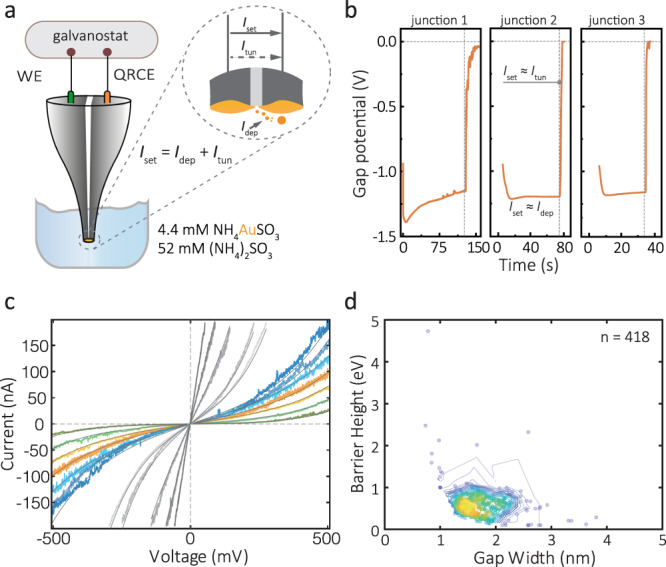
Electrochemical fabrication and characterisation of the QMT probes. **a** Gold is deposited via electrochemical deposition at the tip of two coplanar carbon electrodes. The two carbon electrodes were used as working electrode (WE) and quasi-reference counter electrode (QRCE), respectively. A constant preset current, *I*_set_, of 20 pA–10 nA was typically used. Inset: Representation of the working electrodeposition principle. At a chosen *I*_set_, gold atoms are deposited onto the WE surface until a tunnelling junction is formed. **b** Typical chronopotentiometric response during the electrodeposition process with different preset currents of *I*_set_: 20 pA, 100 pA and 1 nA. The electroplating solution contains 4.4 mM NH_4_AuSO_3_ and 52 mM (NH_4_)_2_SO_3_. **c** Tunnelling current−bias (*I*–*V*) measurement of the resulted QMT probes in air at room temperature (297 K). The solid curves in **c** are the data fits using the Simmons model. It all cases the goodness of fit based on *χ*^2^ was on the order of 10^−8^. **d** Density scatter plot of the gap width of 418 probes as a function of barrier height (density: high in yellow, low in navy blue). Both the gap width and barrier height were obtained from the Simmons model for QMT probes measured in air.

Gold was subsequently electrochemically deposited on the carbon nanoelectrodes in a bipotentiostatic configuration using a two-step process to decrease the electrode gap to within the QMT regime. The first step involves fast chronoamperometry to initiate deposition of gold on the carbon nanoelectrodes (Supplementary Figs. 3 and 4). This is followed by a slower, self-terminating chronopotentiometric step with tunnelling feedback control (Fig. 2a)^22,32^. A constant preset current *I*_set_ was applied between the nanoelectrodes, with one being a quasi-reference counter electrode (QRCE), and the other a working electrode (WE), on which the gold deposition occurred. The preset current *I*_se*t*_ consists of a deposition (Faradaic) component *I*_dep_ and a tunnelling component *I*_tun_, such that *I*_set_ = *I*_dep_ + *I*_tun_. For large gaps that are not in the tunnelling regime, *I*_tun_ ~ 0 and *I*_set_ *~* *I*_dep_. The *I*_set_, therefore, drives the deposition and as a result, the gap between the electrodes decreases. When the gap width decreases to within the tunnelling regime, *I*_tun_ dominates leading to a decrease in *I*_dep_ and hence also slowing of the electrodeposition. When *I*_tun_ approaches *I*_set_, *I*_dep_ approaches zero and the electrodeposition self-terminates. Using a galvanostatic mode where *I*_set_ is held constant, the process can be monitored in real time by measuring the decrease in the applied voltage, *V*_gap_ (Fig. 2b). In our experiments, we used *I*_set_ ranging from 20 pA to 10 nA, which exhibited very similar *V*_gap_–time curves. In total, 650 probes were fabricated, and approximately 85% of all probes resulted in self-terminating tunnelling electrodes. The remaining 15% either had an unstable current or did not demonstrate a decrease of *V*_gap_ over time. The presence of deposited gold was confirmed using a range of techniques including cyclic voltammetry (CV), scanning electron microscopy (SEM), scanning transmission electron microscope (TEM)-energy dispersive spectrometry (STEM–EDS), dark-field microscopy (Fig. 1b and Supplementary Figs. 5–8). The total gold deposit had an effective radius of 340 ± 180 nm (*n* = 10) as determined by CV using hexaammineruthenium(III) chloride (Ru(NH_3_)_6_Cl_3_) as a redox probe, which was significantly larger than the initial size of the carbon electrodes (30 ± 8 nm) (*n* = 10); however, it should be noted that these calculated values are only indicative, as they are based on simplifications in the modelled limiting current, see further discussion in Supplementary Section 2.1. Both dark-field microscopy and STEM–EDS revealed gold was deposited outward from the tip (Fig. 1b (iv and v)).

The final step performed to obtain stable QMT nanoprobes was to immerse the freshly fabricated electrode gaps for 12–48 h in ultrapure deionised water (with a resistivity of 18.2 MΩ cm). After prolonged water exposure, we observed that an average 55% decrease in conductance (*n* = 65), consistent with the widening of the tunnelling gaps (Supplementary Fig. 9). The probes were found to exhibit consistent *I*–*V* characteristics over longer periods (over several days), unlike those fabricated using only electrodeposition. The effective change of gap width can be potentially attributed to the surface diffusion of gold atoms in order to minimise the total interfacial free energy at a fixed volume^33^.

The functionality of the QMT junctions was characterised experimentally by recording *I*–*V* curves and fitting the data to the Simmons model, which can be used to approximate the gap widths, tunnelling barrier height, and tunnelling area (Fig. 2c, d and Supplementary Section 3). Figure 2c shows representative *I*–*V* curves for QMT gaps with large (in colour) and small width (grey), which were fit using the Simmons tunnelling model. In most of the QMT probes, distinct sigmoidal tunnelling behaviour was observed as proposed by the model (Supplementary Fig. 10). Gaps with a smaller width typically have a steeper slope and exhibit more linear shape, especially when the gap width approaches the quantum point contact (with a conductance close to *G*_0_ = 77.5 µS). In total, 418 standalone probes were fabricated with gap width ranging from sub-nm to >3 nm, with an average gap width of 1.6 ± 0.6 nm (Fig. 2d and Supplementary Fig. 11). To further confirm that the fabricated QMT junctions were functional, *I*–*V* curves were measured in media with different barrier height including air, DI water, hexane, and dimethyl sulfoxide (DMSO) (Supplementary Fig. 12a). To exclude interference from cross-contamination, we randomised the sequence at which the solvents were measured. Effective barrier heights were calculated to be 0.37 ± 0.21 eV for DI water, 0.78 ± 0.14 eV for DMSO and 0.97 ± 0.21 eV for hexane, with the values being in agreement with literature^23,24,34^. In air, a barrier height of 1.04 ± 0.83 eV was calculated from the experimental data, and the large variation was attributed possible condensation of water vapour in the gap. Control experiments were also performed with junctions that were bridged (short-circuited electrodes), and those did not exhibit *IV* dependence on the media used (Supplementary Fig. 12b).

### QMT detection of single-stranded DNA and mononucleotides

**Fig. 3:**
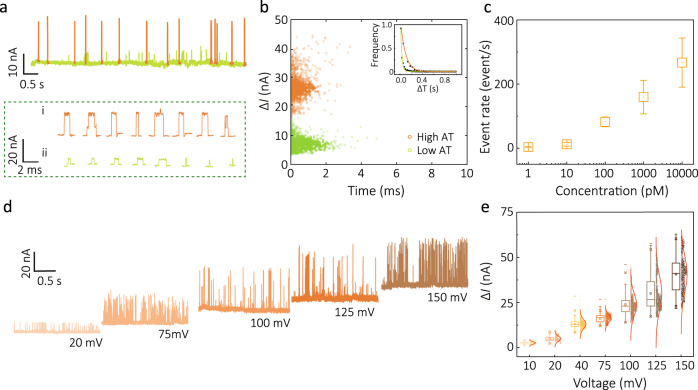
QMT detection of single-stranded DNA. **a** Representative tunnelling current–time trace for 100 pM poly-A20 DNA using a QMT probe with a gap width of ~1.7 nm at an applied bias of 100 mV. Transient current increase indicates a single DNA molecule residing between the tunnelling junction. Both high amplitude (orange) and low amplitude transients (green) are visible above the baseline current. **b** Scatter plots for transient time vs change in current for both high amplitude (orange) and low amplitude transients. The inset shows a histogram for the time between events for both classes of transients, respectively, which could be fitted as single exponential distribution, consistent with single-molecule events. **c** Plot of event rate of the high amplitude transients events vs analyte concentrations (1 pM, 10 pM, 100 pM, 1 nM, and 10 nM). **d** Representative tunnelling current traces for the same QMT probe under different bias in the presence of 100 pM poly-A20 DNA. **e** Histogram and **f** statistics analysis of peak current for high amplitude transients at a different bias (10–150 mV). All error bars represent 1 standard deviation from the mean.

Optimisation and characterisation of the QMT probes for single-molecule detection were initially performed using single-stranded DNA with 20A bases (poly-A20) in 1 mM phosphate buffer (PBS, pH = 7.4) (Fig. 3, Supplementary Section 4 and Supplementary Movie 1). QMT probes with a gap width of 1.8 ± 0.3 nm were used, providing a good balance between molecule size and gap width. With the addition of the analyte to the bath, characteristic spike-like current transients were observed, which was attributed to single molecules coming into close proximity, interacting, or bridging the gap (Supplementary Movie 1 and Supplementary Fig. 13). The high stability of the QMT junctions meant that repeated experiments could be performed with the same probe over more than 24 h (Supplementary Fig. 14). Both the amplitude of the spikes and the amplitude of the background current varied significantly between different probes; however, the relative amplitude of the transients was consistent for probes with the similarly sized gaps.

The amplitude of the transient was defined by the maximum current value of each peak subtracted from the base level (*I*_p_), and molecular conductance (∆*G*) was calculated using *I*_p_/*V*_bias_, where *V*_bias_ is the voltage bias applied between the electrodes. A close look at each transient revealed the presence of multiple levels in the tunnelling (Fig. 3a). Low amplitude transients with a mean peak conductance of 76 ± 20 nS (*n* = 1545) were attributed to DNA adsorbing/desorbing to a single QMT electrode. This interaction is likely caused by a modulation in the capacitance at the electrode–solution interface, or a local change in the polarisation of the medium near the gap^13^. The higher amplitude transients with mean peak conductance 267 ± 49 nS (*n* = 1017) were likely caused by the DNA bridging and binding to both electrodes, followed by desorption. The width of each transient (*t*_d_) for both low and high amplitude transients represented the trapping duration and was determined to be *t*_d_ = 0.39 ± 0.55 ms for low and 0.44 ± 0.45 ms for high amplitude, respectively. These transients were very distinct and the time between single events followed an exponential distribution, as would be expected for a stochastic process (Fig. 3b, inset). Voltage (20–150 mV) and concentration (10 pM–10 nM) dependence was also explored. With increasing concentration, the event rate increased from 12 ± 6 events/s at 10 pM to 267 ± 77 events/s at 10 nM (Fig. 3c and Supplementary Fig. 13). As for high amplitude transients, a predominately linear current dependence on voltage (10–150 mV) was observed with a slope of 233 ± 19 nS (Fig. 3d, e and Supplementary Fig. 15).

**Fig. 4:**
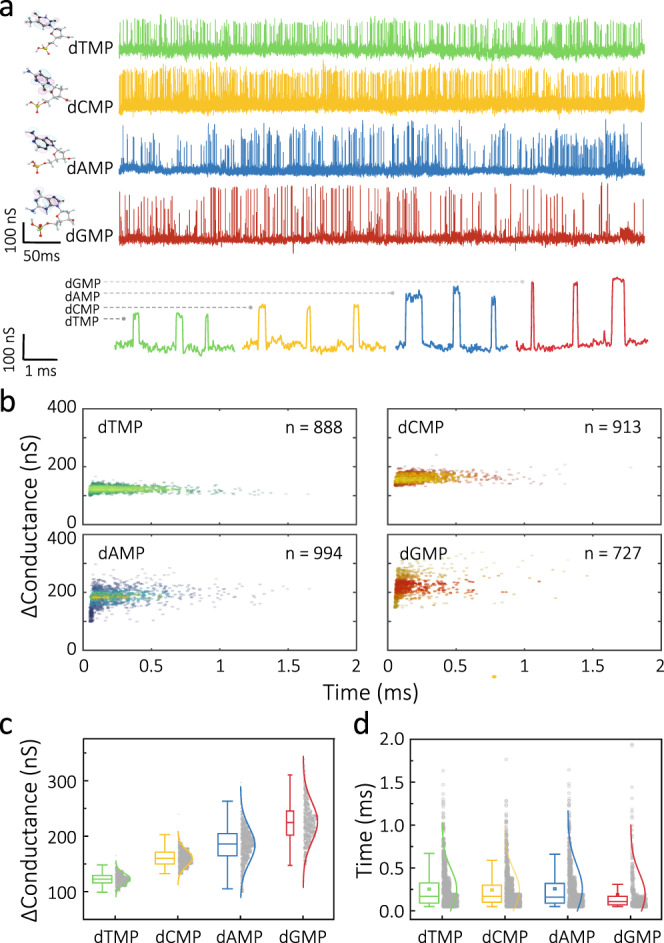
QMT detection of single nucleotides. **a** Conductance vs time trace for four deoxymononucleotides: dTMP (green), dCMP (orange), dAMP (blue) and dGMP (red). Data were acquired in 1 mM PBS (pH 7.4) containing 100 pM nucleotides, at 50 mV. The gap width was ~1.1 nm. **b** Scatter plot of the transients for each mononucleotide. **c**, **d** Normalised bar chat of the conductance and dwell time for each nucleotide. Mean values are presented as means ± SD, as shown in Supplementary Table 2. ***P < 0.001 (two-tailed *P* value), determined by Student’s *t*-test.

In addition, we performed tunnelling conductance measurements on single mononucleotides including deoxyadenosine 5′-monophosphate (dAMP), thymidine 5′-monophosphate (dTMP), deoxycytidine 5′-monophosphate (dCMP) and deoxyguanosine 5′-monophosphate (dGMP) (Fig. 4a). This was done by performing chronoamperometric measurements using ~1.1 nm gap at a bias voltage of 50 mV. This gap size was chosen as it is slightly larger than the dimensions of a single nucleotide (<1 nm)^16,35^. Each analyte type produced current transients that corresponded to specific peak conductance (∆*G*), with dGMP (240 ± 36 nS) > dAMP (180 ± 33 nS) > dCMP (161 ± 5 nS) > dTMP (120 ± 10 nS) (Fig. 4b, c). This trend was preserved in probes with different gap widths (e.g. 0.7 and 1.9 nm) (Supplementary Figs. 16–18 and Supplementary Table 2). We also performed tunnelling detection of a two-base mixture containing equimolar amounts (100 pM) of dTMP and dGMP (Supplementary Fig. 19), revealing two distinct peaks. These observations are in good agreement with the results obtained for single nucleotides discrimination using break junctions with a 0.6 nm gap^35,36^. Although the actual electron transport mechanism across mononucleotides remains an open question, the large increase in tunnelling current likely relies on the strong electronic coupling between the analyte and the electrodes. Changes in mononucleotide conductances can in part be interpreted qualitatively by the differences in the energy level of the highest occupied molecular orbital (HOMO). Each mononucleotide has a different local electronic density of states with a spatial extent owing to distinct chemical composition. For example, dGMP showed the highest conductance among other mononucleotides because of its HOMO level is aligned closer to the Fermi level of the QMT electrodes^13,16,35^. Owing to the differences in HOMO energies, the four nucleotides can be distinguished by their molecular conductance. In principle, this methodology can be further applied to obtain further information such as orientation and rotation of the molecule^16,36^.

In all cases, the mean event times had similar values, which is expected for molecules with similar shape and size, as can be seen in Fig. 4d. Although there were slight variation in the average event times between mononucleotides due to the different binding affinity to gold surfaces^37^, these variations were within experimental error.

### QMT detection of proteins

**Fig. 5:**
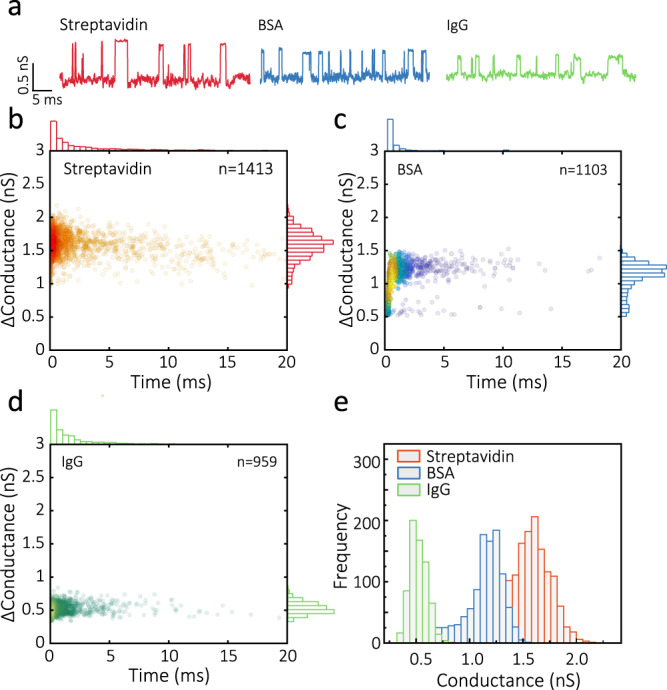
QMT detection of streptavidin, BSA and IgG. **a** Representative conductance–time traces for a solution consisting of 10 nM of proteins using a tunnelling junction with a gap width of ~1.8 nm and an applied bias of 100 mV. **b**–**d** Scatter plots of change in conductance versus duration for transients associated with streptavidin, BSA, and IgG. **e** Normalised histogram of the change in conductance for the three proteins highlighting a clear difference in amplitude of the transients.

The detection and analysis of proteins at the single-molecule level are particularly important, especially at low concentrations. However, unlike DNA, proteins and peptides have heterogeneous charge distribution, making detection more challenging. Recently tunnelling detection of proteins has been experimentally shown using or STM-based recognition tunnelling platforms^18,38,39^. We confirmed the suitability of our probes for the detection of proteins, using a panel of three protein analytes with different molecular weight and charge, including streptavidin (SA), bovine serum albumin (BSA) and immunoglobin G (IgG) (Fig. 5). Although the gap width (~1.8 nm) was smaller than the size of the proteins, characteristic tunnelling signals could be obtained. Similar to the case of DNA and nucleotides, when introduced in the bath, the protein analytes lead to the observation of amplitude transients (Fig. 5a). The data are summarised in scatter plots for the conductance and event times for the three proteins at 100 mV bias (Fig. 5b–d). Typically, the width of the dwell time distribution spanned more than three orders of magnitude ranging from 200 μs to 20 ms, with the majority of events occurring on faster time scales (sub-1 ms). The small fraction of longer-lived events is likely due to a variation in junction electrode-molecule binding affinity associated with the diverse molecular conformations of mobile proteins in the electrode gaps. However, well-defined conductance peaks were observed, indicating a preferred conformational arrangement of the protein in the tunnelling junction. Δ*G* for each protein (Fig. 5e) exhibited significantly different values (1.56 ± 0.19 nS for SA, 1.11 ± 0.21 nS for BSA and 0.52 ± 0.08 nS for IgG under 100 mV bias). According to previous findings, non-redox proteins might feature a high electronic conductance when measured using ligand-functionalised tunnelling electrodes^14,35,36^. Interestingly, we used the QMT probes without ligands or other good chemical modifications, and a reasonably high conductance could still be observed. Considering that the probes can be fabricated with a tuneable tunnelling gap width, we believe that these probes can be valuable in studying protein tunnelling and performing protein analysis using direct electrical readout^40^.

### QMT detection using DEP trapping at ultralow concentrations

As shown above, our probes work well at pM and nM concentrations for DNA and proteins, respectively. This is in part facilitated by the sharp (aspect ratio, ~5 × 10^5^) and conical probe geometry that allows analyte to access the junction from almost all directions compared to planar tunnelling devices. Compared to scanning tunnelling scanning probes, the standalone probe design integrating a tunnelling junction at the probe tip also offers unobstructed analyte access, as there is no requirement for our probes to be within a tunnelling distance of a conductive substrate to function. Generally, in tunnelling platforms, analyte transport tends to be diffusion-limited or relies on surface adsorption. By definition, the electrode gaps are highly confined, and it is energetically unfavourable for the analyte to transit the gaps, which in turn result in low detection rates. In some cases, event rates can be improved at low concentrations by incorporating ligands on the electrode surface^13^; however, the limit of detection can also be enhanced by actively transporting molecules to the probe.

**Fig. 6:**
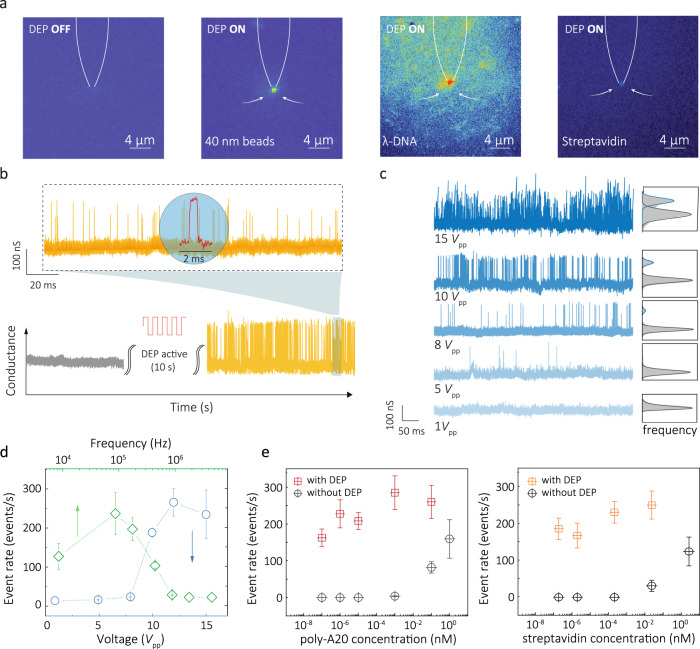
Incorporation of DEP trapping in the QMT sensors for the molecular detection at ultralow concentration. **a** Fluorescence imaging of the nanoprobe under application of an AC DEP trapping voltage for fluorescently labelled beads, lambda DNA and proteins. (i) control experiment without applied AC voltage. **b** Tunnelling current traces of a typical DEP (*V*_pp_ 10 V, *f*_AC_ 100 kHz, 10 s) pre-concentration/detection cycle for detection of 1 fM poly-A20. **c** Typical conductance time traces for 1 fM poly-A20 at different AC trapping voltages. Normalised histograms of the conductance and AC frequency *f*_AC_ = 100 kHz, and trapping time of 10 s before performing tunnelling measurements at an applied DC bias of 50 mV. **d** Analysis of improvement in event rates as a function of DEP voltage and frequency. Event rate vs AC voltage was measured at *f*_AC_ 100 kHz, Event rate vs AC frequency was measured at AC voltage of 10 *V*_pp_. **e**, **f** Event rate for the detection of poly-A20 and streptavidin when compared to performing measurements with and without DEP. All error bars represent 1 standard deviation from the mean.

Here we show that our QMT probes can also be used in combination with AC based DEP trapping (Fig. 6). Since the probe consists of two individually addressable nanoelectrodes with gaps in the nm regime, similar to a single-molecule nanotweezer, it is ideally suited for trapping single DNA and protein molecules^28–30^. Briefly, when an AC field is applied across the electrodes, it generates exceptionally high electric field gradients, thus enabling the trapping of single molecules^28–30^. When the probe was used in DEP trapping mode, both electrodes served as the working electrode. Typically, the DEP trapping and QMT experiments were performed sequentially (i.e. trapping followed by tunnelling detection), which temporarily increased the local analyte concentration, prior to detection. Unfortunately, measurements could not be performed in parallel due to the significant increase in low-frequency noise typically associated with conductance fluctuations (flicker noise) and a mild increase in higher frequency noise attributed to capacitance when an AC field was applied. Throughout all experiments, current–voltage curves were generated to ensure consistent tunnelling behaviour (Supplementary Fig. 20).

To demonstrate and characterise the DEP trapping, fluorescently labelled beads (diameter of 40 nm), λ-DNA and SA were used for a detailed description of the experiment set-up (see Supplementary Figs. 21 and 22). Key parameters which were optimised include the AC frequency (*f*_AC_) and the AC peak-to-peak voltage (*V*_pp_). As can be seen in Fig. 6a, once the AC field was turned on, the fluorescently labelled analytes would localise and surround the tip region of the probe. For example, for YOYO-1-labelled λ-DNA, applying an AC voltage (*V*_pp_ = 10 V, *f*_AC_ = 100 kHz) resulted in the accumulation of DNA molecules around the probe (Supplementary Fig. 22 and Supplementary Movie 2) with an increase in fluorescence intensity being observed over time. When the AC voltage was turned off, the fluorescent intensity at the probe tip decreased due to the analyte diffusing away. In contrast, no significant fluorescence change was observed at the tip in the absence of an AC voltage, minimising the possibility of any non-specific adsorption of DNA molecules onto the electrodes.

Conductance–time traces pre and post trapping are shown for 1 fM poly-A20 DNA (Fig. 6b). DEP trapping took place for 10 s at *f*_AC_ 100 kHz and *V*_pp_ 10 V followed by immediate turning on of the DC voltage for QMT experiments. At such low concentrations, no significant tunnelling events were observed without DEP trapping. As expected, the trapping efficiency was directly dependent on applied peak-to-peak voltage (*V*_pp_) and frequency (*f*_AC_), where a clear increase in event rates is seen under optimal conditions (Fig. 6c, d and Supplementary Fig. 23). At higher *V*_pp_ (e.g. 15 V) or low *f*_AC_ (e.g. 10 kHz), the probes became increasingly unstable. This likely stems from Faradaic processes or heating taking place at the electrode surface. A significant advantage of using DEP is that exceptionally low analyte concentrations can be used. For example, event rates can be significantly enhanced as is shown for poly-A20 and proteins with and without DEP (Fig. 6e, f). Impressively this method allowed for a ~5 order increase in analyte detection rates and the possibility to perform QMT detection down to sub-fM (<10^−15^ mol/L), e.g., 0.1 fM for poly-A20 and 0.15 fM for SA. However, the event rate at the lowest concentrations probed (e.g., <10^6^ molecules in 1 mL for 0.1 fM poly-A20) is very high (>200 events/s) and does not have a significant dependence on concentration. This is likely due to once molecules are trapped, they will localise and accumulate around the tips and have a much higher probability of being recaptured and interact with the tunnelling gap. As a result, it is likely that the same molecules can be detected multiple times.

To conclude, we have demonstrated a robust, reproducible and controllable methodology for the fabrication of QMT probes and achieved high sensitivity single-molecule tunnelling detection by combining with DEP. The probe, consisted of two nanoelectrodes with gaps fabricated using a feedback mechanism, were fabricated from dual-barrel quartz theta capillaries. The tip of the nanopipette was used as the basic architecture of coplanar carbon electrodes by pyrolytic deposition of carbon. Under a feedback-controlled electrochemical deposition process, the interelectrode distance can be accurately tuned for specific gap widths.

We demonstrated that these probes could be used to efficiently operate in solution environments and detect single molecules via electron tunnelling ranging from mononucleotides, DNA oligomers, to a range of proteins with different size. The integrated tunneling junction design and the high aspect ratio geometry of the probe allowed for analyte to access the junction from almost all directions, enabling efficient analyte transport. We showed that these probes could be combined with DEP trapping to efficiently attract molecules towards the tunnelling electrodes, enabling active analyte transport to the tunnelling sensor and analyte pre-concentration. The latter allows for up to five orders of magnitude increase in event detection rates and sub-femtomolar sensitivity. This regime is also currently inaccessible using state-of-the-art tunnelling technology. To our knowledge, this is the first example of dynamic control of molecular transport that has been used in any tunnelling system.

A unique feature of the probes is their standalone design, which does not require a conductive substrate to operate, unlike other techniques such as scanning tunnelling microscopy. This opens the door to a range of applications, including the study of non-conductive and soft matter interfaces that are currently inaccessible using state-of-the-art tunnelling technology.

## Methods

### Materials

2′-Deoxyadenosine 5′-monophosphate (dAMP, H form, catalogue No. D6375; Sigma grade, 98–100%), 2′-deoxycytidine 5′-monophosphate (dCMP, H form, catalogue No. D7750; Sigma grade, 95%), 2′-deoxyguanosine 5′-monophosphate (dGMP, sodium form, catalogue No. D9500; HPLC grade, g99%), and thymidine 5′-monophosphate (dTMP, disodium form, catalogue No. T7004; Sigma grade, g99%) were used as received. The 10 kbp DNA and λ-DNA (both 500 µg/mL) were purchased from New England Biolabs. DNA oligomers were synthesised and characterised by Integrated DNA Technologies and used without further purification. All chemicals used in the device fabrication and electrochemical characterisation were bought from Sigma Aldrich apart from the plating solution.

### Fabrication of double-barrel carbon nanoelectrodes

The carbon nanoelectrodes were fabricated according to the methods used for fabricating nanoscale tweezers (Supplementary Fig. 3). Briefly, the double-barrel theta quartz capillaries were first pulled by a two-line programme using a P-2000 laser puller (Sutter Instrument). Next, butane was passed through the pulled nanopipettes and heated, under an argon atmosphere to pyrolytically deposit carbon inside the pipette. After carbon deposition, the carbon nanoelectrodes consisted of two coplanar semi-elliptical electrodes encapsulated in quartz, which were separated by a quartz septum*.* All of the nanoelectrodes were fabricated immediately before experiments, unless noted otherwise, and stored in a sealed Petri dish until used, to minimise any contamination.

### Imaging and characterisation of the tunnelling junctions

The tips of the QMT probes were prepared by focused ion beam cutting (FIB, Quanta 3D FEG) with a Ga^+^ ion accelerating voltage of 30 kV. The microstructure and element distribution were studied by a TEM at 200 kV (Tecnai G2 F20, FEI), equipped with energy dispersive X-ray spectroscopy. *I*–*V* measurement was performed to characterise the gap distances and barrier height. Each of the tips was first removed from deionised water. The tip of the nanopipettes was also imaged by A Zeiss Gemini Sigma 300 field emission gun scanning electron microscope, at an acceleration voltage of 5 kV. The *I*–*V* curves were fit using the Simmons Model using custom-written software (see Supplementary Section 3).

### DEP trapping

The QMT probe was inserted into the solution with different target concentrations (0.1 fM–10 nM for poly-A20 and 0.15 fM–1.5 nM for streptavidin respectively). The experimental configuration is shown in Supplementary Fig. 19. Molecular trapping was initiated by turning on the AC voltage for the desired time period (10–20 s). This was followed by switching off and applying the DC voltage for tunnelling measurements.

### Data analysis

All tunnelling current measurements were recorded using a MultiClamp 700B operated in voltage-clamp mode. The recorded current signal was filtered using 4-pole Bessel filter at 10 kHz and digitised using an Axon Digidata 1550B. Data analysis was performed using a custom-written MATLAB code developed in-house.

## Supplementary information

Supplementary Information

Description of Additional Supplementary Files

Supplementary Movie 1

Supplementary Movie 2

## Data Availability

The data that support the plots within this paper and other findings of this study are available from the corresponding author upon reasonable request.

## References

[1] Michalet X, Weiss S, Jager M (2006). Single-molecule fluorescence studies of protein folding and conformational dynamics. Chem. Rev..

[2] Di Ventra M, Taniguchi M (2016). Decoding DNA, RNA and peptides with quantum tunnelling. Nat. Nanotechnol..

[3] Restrepo-Perez L, Joo C, Dekker C (2018). Paving the way to single-molecule protein sequencing. Nat. Nanotechnol..

[4] Albrecht T (2012). Electrochemical tunnelling sensors and their potential applications. Nat. Commun..

[5] Sze JYY, Ivanov AP, Cass AEG, Edel JB (2017). Single molecule multiplexed nanopore protein screening in human serum using aptamer modified DNA carriers. Nat. Commun..

[6] Jaculbia RB (2020). Single-molecule resonance Raman effect in a plasmonic nanocavity. Nat. Nanotechnol..

[7] Xue, L. et al. Solid-state nanopore sensors. *Nat. Rev. Mater*. **5**, 931–951 (2020).

[8] Xin N (2019). Concepts in the design and engineering of single-molecule electronic devices. Nat. Rev. Phys..

[9] Fanget A (2014). Nanopore integrated nanogaps for DNA detection. Nano Lett..

[10] Simmons JG (1963). Generalized formula for the electric tunnel effect between similar electrodes separated by a thin insulating film. J. Appl. Phys..

[11] Shapir E (2008). Electronic structure of single DNA molecules resolved by transverse scanning tunnelling spectroscopy. Nat. Mater..

[12] Li Y (2018). Detection and identification of genetic material via single-molecule conductance. Nat. Nanotechnol..

[13] Pang P (2014). Fixed-gap tunnel junction for reading DNA nucleotides. ACS Nano.

[14] Huang S (2010). Identifying single bases in a DNA oligomer with electron tunnelling. Nat. Nanotechnol..

[15] Zhao Y (2014). Single-molecule spectroscopy of amino acids and peptides by recognition tunnelling. Nat. Nanotechnol..

[16] Tsutsui M, Taniguchi M, Yokota K, Kawai T (2010). Identifying single nucleotides by tunnelling current. Nat. Nanotechnol..

[17] Ohshiro T (2014). Detection of post-translational modifications in single peptides using electron tunnelling currents. Nat. Nanotechnol..

[18] Zhang B (2019). Role of contacts in long-range protein conductance. Proc. Natl Acad. Sci. USA.

[19] Chang S (2009). Tunnelling readout of hydrogen-bonding-based recognition. Nat. Nanotechnol..

[20] Park H, Lim AKL, Alivisatos AP, Park J, McEuen PL (1999). Fabrication of metallic electrodes with nanometer separation by electromigration. Appl. Phys. Lett..

[21] Li CZ, He HX, Tao NJ (2000). Quantized tunneling current in the metallic nanogaps formed by electrodeposition and etching. Appl. Phys. Lett..

[22] Xiang J (2005). A controllable electrochemical fabrication of metallic electrodes with a nanometer/angstrom-sized gap using an electric double layer as feedback. Angew. Chem. Int. Ed. Engl..

[23] Ivanov AP, Freedman KJ, Kim MJ, Albrecht T, Edel JB (2014). High precision fabrication and positioning of nanoelectrodes in a nanopore. ACS Nano.

[24] Ivanov AP (2011). DNA tunneling detector embedded in a nanopore. Nano Lett..

[25] Jia C (2016). Covalently bonded single-molecule junctions with stable and reversible photoswitched conductivity. Science.

[26] Healy K (2012). Fabrication and characterization of nanopores with insulated transverse nanoelectrodes for DNA sensing in salt solution. Electrophoresis.

[27] Zhan C (2020). Single-molecule plasmonic optical trapping. Matter.

[28] Barik A (2017). Graphene-edge dielectrophoretic tweezers for trapping of biomolecules. Nat. Commun..

[29] Freedman KJ (2016). Nanopore sensing at ultra-low concentrations using single-molecule dielectrophoretic trapping. Nat. Commun..

[30] Nadappuram BP (2019). Nanoscale tweezers for single-cell biopsies. Nat. Nanotechnol..

[31] Cadinu P (2017). Single molecule trapping and sensing using dual nanopores separated by a zeptoliter nanobridge. Nano Lett..

[32] Xiang J, Liu B, Liu B, Ren B, Tian Z-Q (2006). A self-terminated electrochemical fabrication of electrode pairs with angstrom-sized gaps. Electrochem. Commun..

[33] Xia YN, Xiong YJ, Lim B, Skrabalak SE (2009). Shape-controlled synthesis of metal nanocrystals: simple chemistry meets complex physics?. Angew. Chem. Int. Ed..

[34] Prokopuk N, Son K-A, Waltz C (2007). Electron tunneling through fluid solvents. J. Phys. Chem. C.

[35] Furuhata T (2019). Highly conductive nucleotide analogue facilitates base-calling in quantum-tunneling-based DNA sequencing. ACS Nano.

[36] Tsutsui M (2011). Electrical detection of single methylcytosines in a DNA oligomer. J. Am. Chem. Soc..

[37] Tan LH (2015). Mechanistic insight into DNA-guided control of nanoparticle morphologies. J. Am. Chem. Soc..

[38] Zhang, B. et al. Observation of giant conductance fluctuations in a protein. *Nano Futures* **1**, 035002 (2017). 10.1088/2399-1984/aa8f91 PMC5851656 29552645

[39] Zhang B, Lindsay S (2019). Electronic decay length in a protein molecule. Nano Lett..

[40] Lindsay S (2020). Ubiquitous electron transport in non-electron transfer. Proteins.

