# Supplementary material for: Combined quantum tunnelling and dielectrophoretic trapping for molecular analysis at ultra-low analyte concentrations
Source: Nat Commun. 2021 Feb 10;12:913. doi: 10.1038/s41467-021-21101-x (PMC7876030; doi:10.1038/s41467-021-21101-x)
Supplement: Supplementary file 1 — Supplementary Information [file 41467_2021_21101_MOESM1_ESM.pdf]

Combined quantum tunnelling and dielectrophoretic trapping for molecular analysis at ultra-low analyte concentrations

Longhua Tang,[1,2,3]* Binoy Paulose Nadappuram,[2] Paolo Cadinu,[2] Zhiyu Zhao,[4] Liang Xue,[2] Long Yi,[2] Ren Ren,[2] Jiangwei Wang,[4] Aleksandar P. Ivanov,[2]* Joshua B. Edel [2]*

[1] State Key Laboratory of Modern Optical Instrumentation, College of Optical Science and Engineering; International Research Center for Advanced Photonics, Zhejiang University, Hangzhou 310027, China
[2] Department of Chemistry, Molecular Science Research Hub, Imperial College London, White City Campus, 80 Wood Lane, London, W12 0BZ, UK
[3] Innovation Institute for Artificial Intelligence in Medicine, Zhejiang University, Hangzhou 310018, China
[4] Center of Electron Microscopy and State Key Laboratory of Silicon Materials, School of Materials Science and Engineering, Zhejiang University, Hangzhou 310027, China

* Corresponding authors: lhtang@zju.edu.cn, alex.ivanov@imperial.ac.uk, joshua.edel@imperial.ac.uk

Table of Contents

Supplementary Note 1 Fabrication of the QMT probes........ 3

Supplementary 1.1 Overview of the workflow of the fabrication of tunnelling devices from the dual-barrel quartz nanocapillary........ 3

Supplementary 1.2 Fabrication of dual-barrel nanopipettes........ 3

Supplementary 1.3 Fabrication of carbon nanoelectrodes by pyrolysis of butane........ 4

Supplementary 1.4 Electrochemical etching of carbon nanoelectrodes........ 4

Supplementary 1.5 Gold pre-deposition on carbon nanoelectrodes........ 5

Supplementary Note 2 Characterisation of QMT probes........ 7

Supplementary 2.1 Electrochemical characterisation of QMT probes........ 7

Supplementary 2.2 Optical and structural characterisation of QMT probes........ 8

Supplementary 2.3 Water reshaping of gold-deposited nanoelectrodes over long time periods........ 9

Supplementary Note 3 Functionality characterisation of QMT probes........ 9

Supplementary Note 4 QMT detection of different molecules........ 18

Supplementary 4.1 QMT detection of poly-A20 at different concentrations........ 18

Supplementary 4.2 QMT detection of poy-A20 using tunnelling probes with different gap width........ 20

Supplementary Note 5 Dielectrophoretic - tunnelling detection using QMT probes........ 24

Supplementary 5.1 Stability evaluation of QMT probes under DEP trapping........ 24

Supplementary 5.2 Fluorescence imaging and characterisation of QMT probes with DEP trapping........ 24

Supplementary References........ 26

Supplementary Note 1 Fabrication of the QMT probes

Supplementary 1.1 Overview of the workflow of the fabrication of tunnelling devices from the dual-barrel quartz nanocapillary

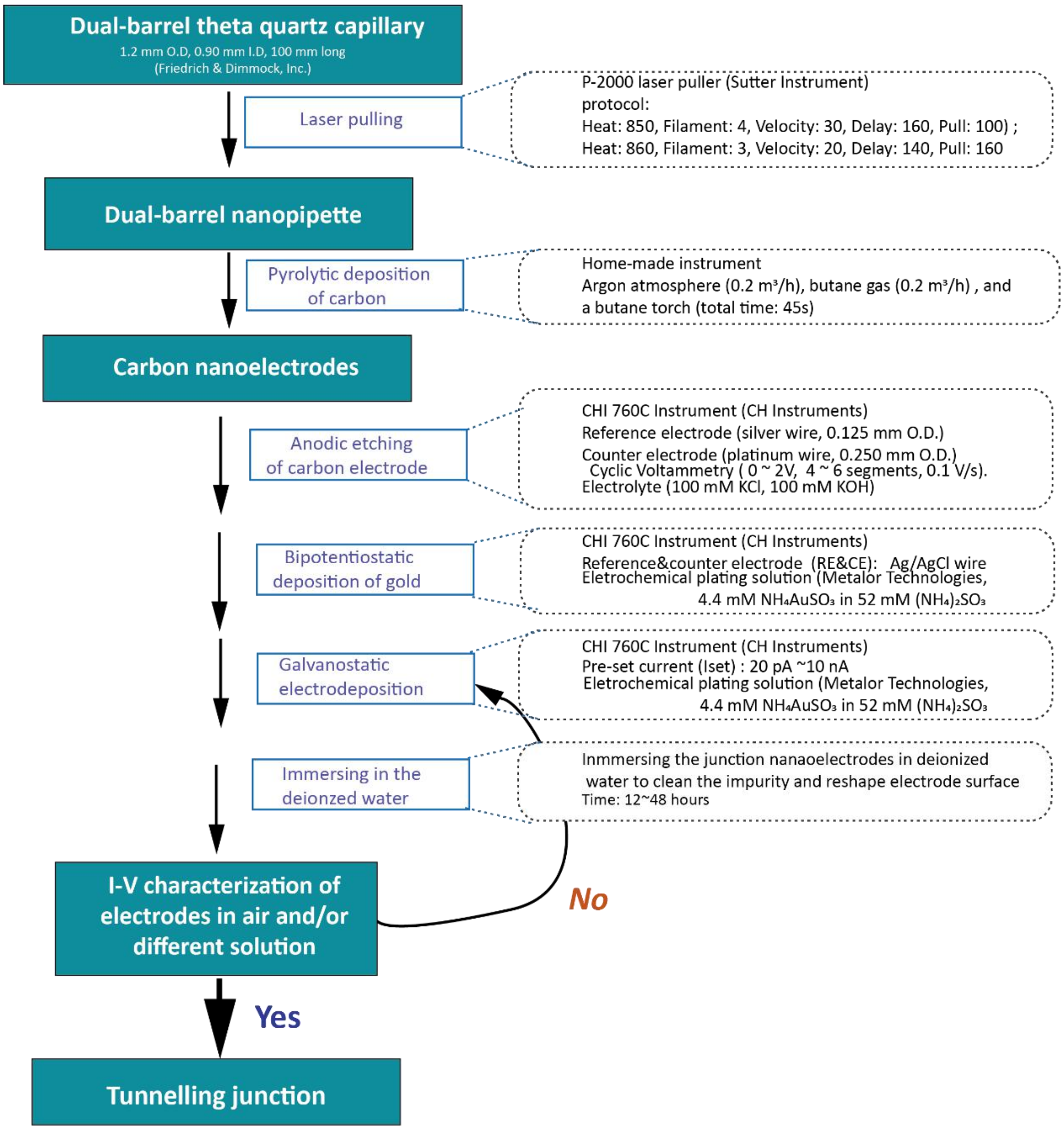

Supplementary Fig. 1| The workflow of the fabrication of QMT probes. These probes were fabricated from theta-shaped dual-barrel quartz capillaries. First, the quartz capillaries were laser pulled to a sharp nanopipette with tip terminating in two closely spaced nanopores separated by a quartz septum. Both barrels of the nanopipette were then filled with carbon by the pyrolytic decomposition of butane, resulting in the generation of two co-planar carbon nanoelectrodes. Finally, gold was electrochemically deposited onto the tips of the nanoelectrodes in a bipotentiostatic configuration using a two-step process to bring the electrode gaps to within the QMT regime. The first step involves fast chronoamperometry to initiate deposition of gold on the etched carbon nanoelectrodes. This is followed by a slower, self-terminating chronopotentiometric step with feedback control to form the tunnelling junction. Finally, stable QMT nanoprobes were immersed in ultrapure deionised water (with a resistivity of 18.2 MΩ cm) for 12–48 hours.

Supplementary 1.2 Fabrication of dual-barrel nanopipettes.

The dual-barrel theta quartz capillaries (Friedrich & Dimmock, Inc., 1.2 mm OD, 0.90 mm ID, 100 mm long) were cleaned in PDC-002 Expanded Plasma Cleaner (Harrick Plasma) for 30 minutes to remove surface contaminants. The cleaned capillaries were then pulled by a P-2000 laser puller (Sutter Instruments) using a custom two-line program (First line; Heat: 850, Filament: 4, Velocity: 30, Delay: 160, Pull: 100. Second-line Heat: 860, Filament: 3, Velocity: 20, Delay: 140, Pull: 160) to form sharp-ended nanopipettes. Dual-barrel nanopipettes with a diameter ranging from 20 nm to 100 nm across each barrel can be fabricated by varying the program parameters. Note that pulling programs are device-specific and are sensitive toward the variation of humidity and temperature.[1, 2]

Supplementary 1.3 Fabrication of carbon nanoelectrodes by pyrolysis of butane.

Schematics of the pyrolytic carbon deposition process is shown in Supplementary Fig. 2a. The sharp dual-barrel nanopipette tip was inserted into argon atmosphere (0.2 m^3/h) and butane gas (0.2 m^3/h) was introduced through the nanopipette via tubing. The tip of the nanopipette was then heated using a butane torch for 10 seconds and was moved along the nanopipette to heat the probe for 30 seconds to pyrolytically deposit carbon from the butane. The nanopipette tip was kept under an argon flow after the pyrolysis until it cooled down (typically 5 – 10 seconds). All the nanopipettes used herein were stored in a sealed Petri dish until use to minimise any contamination. The presence of fully formed carbon electrodes after pyrolytic deposition, and gold deposit after electrodeposition was confirmed by Energy-dispersive X-ray spectroscopy (EDS) maps of the tip using Transmission Electron Microscopy and Energy-dispersive X-ray spectroscopy (STEM-EDS). Both the TEM micrograph (Fig. 2a), the oxygen (Fig. 2c), and the silicon (Fig. 2d) maps are due to the quartz sheath forming the pipette, while the carbon map (Fig. 2b), confirmed the presence of the carbon electrodes at the tip of the nanopipette.[3, 4]

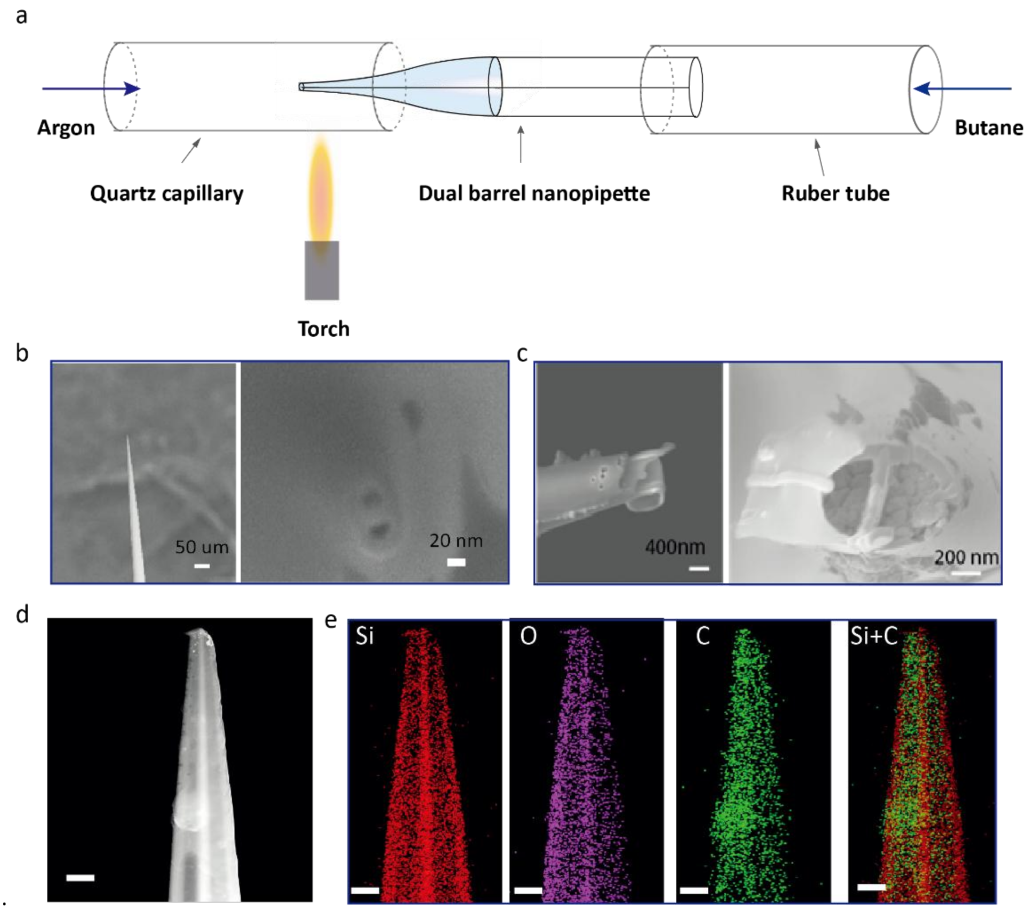

Supplementary Fig. 2| Fabrication and characterisation of carbon electrodes at the tip of the dual barrel nanopipettes. (a) Schematic of the carbon deposition step. Butane was passed through the pulled nanopipette kept in argon atmosphere. The tip of the probe was heated with a butane torch for to pyrolytically deposit carbon from the butane. (b and c) The SEM image (side view and top view) of an intact and damaged nanopipette. From the images, the conical shape of the nanopipette and the carbon-filled nanopore located on the tip can be clearly observed. (d) STEM image of a typical carbon-filled nanopipette and (e) STEM-EDS map of the same nanopipette showing the presence of silicon, oxygen and deposited carbon at the nanopipette tip. Scale bar: 50 nm.

Supplementary Table 1| Percentage elemental composition of a typical carbon-filled nanoelectrode obtained from STEM−EDS elemental analysis.

Elemental	C	O	Si
Content (%)	72.2	15.1	7.8

Supplementary 1.4 Electrochemical etching of carbon nanoelectrodes.

Electrical contact with the carbon electrodes was established by inserting the copper wires (Goodfellow, 0.5 mm

OD) into the carbon barrel. The copper wire inserted into the barrels of the QTM probe. The carbon nanoelectrodes were electrochemically etched in solution (100 mM KCl, 100 mM KOH). The etching process was carried out by cyclic voltammetry (Initial: 0 V, Final: 2 V, Segment: 4 to 6, Sample Interval: 0.1 V/s) using a potentiostat (CHI 760C). Both carbon nanoelectrodes acted as working electrodes (WE). A silver wire (0.25 mm OD) and a platinum wire (0.25 mm OD) respectively were used as the quasi-reference electrode (QRE) and counter electrode (CE). During the electrochemical etching, carbon deposits on the quartz surface were removed and to create concave electrode surface was formed to facilitate electrodeposition of gold onto the carbon and significantly improve the mechanical and stability of the QMT probe.[5, 6] By comparing the etching current density of both electrodes, which reflects the geometry and quality of the carbon nanoelectrodes, we could screen out nanoelectrodes with asymmetrical geometry and small exposed electrode area for the gold deposition afterwards.

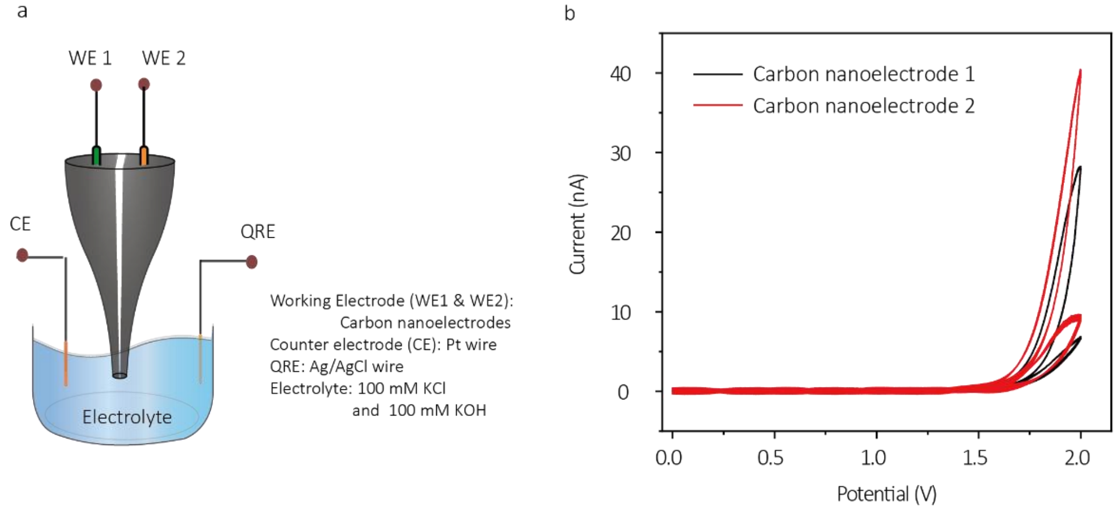

Supplementary Fig. 3| Electrochemical etching of carbon nanoelectrodes. (a) Schematic representation of the electrochemical etching of the carbon nanoelectrodes. The etching process was carried out employing Cyclic Voltammetry (Initial: 0 V, Final: 2 V, Segment: 4~6, Sample Interval: 0.1 V/s) in a 100 mM KOH solution containing 100 mM KCl. (b) typical Cyclic Voltammogram recorded during the electrochemical etching of carbon (scan rate: 100 mV/s).

Supplementary 1.5 Gold pre-deposition on carbon nanoelectrodes.

A bipotentiostatic configuration was used to perform electrodeposition of gold on the carbon electrode (Figure S5a). A diluted ECF64D (Metalor Technologies) plating solution containing 4.4 mM NH_4AuSO_3 in 52 mM $(NH_4)_2SO_3$ was used as the electrolyte and freshly made Ag/AgCl wire (Goodfellow, 0.125 mm OD) was used as the quasi-reference counter electrode (QRE&CE), while the two carbon nanoelectrodes acted as the working electrodes (WE1 and WE2). For electrodeposition of gold, a potential difference of 20 mV was maintained between the two electrodes (1st V: -730 mV, 2nd V: -750 mV) and a current between the electrodes is monitored during the electrodeposition (Figure S5b). To avoid the formation of electrical contact between the two electrodes, the deposition time was limited in 20~50 s. If left longer, an abrupt (normally < 1s) increase in both the current was observed, which is attributed to a sharp rise in tunnelling current when the gap distance was close to tunnelling region (c and d in Figure S5). To avoid the formation of electrical bridge between the nanoelectrode pair, a short deposition time (usually less than 50 seconds) was used for the pre-deposition step.

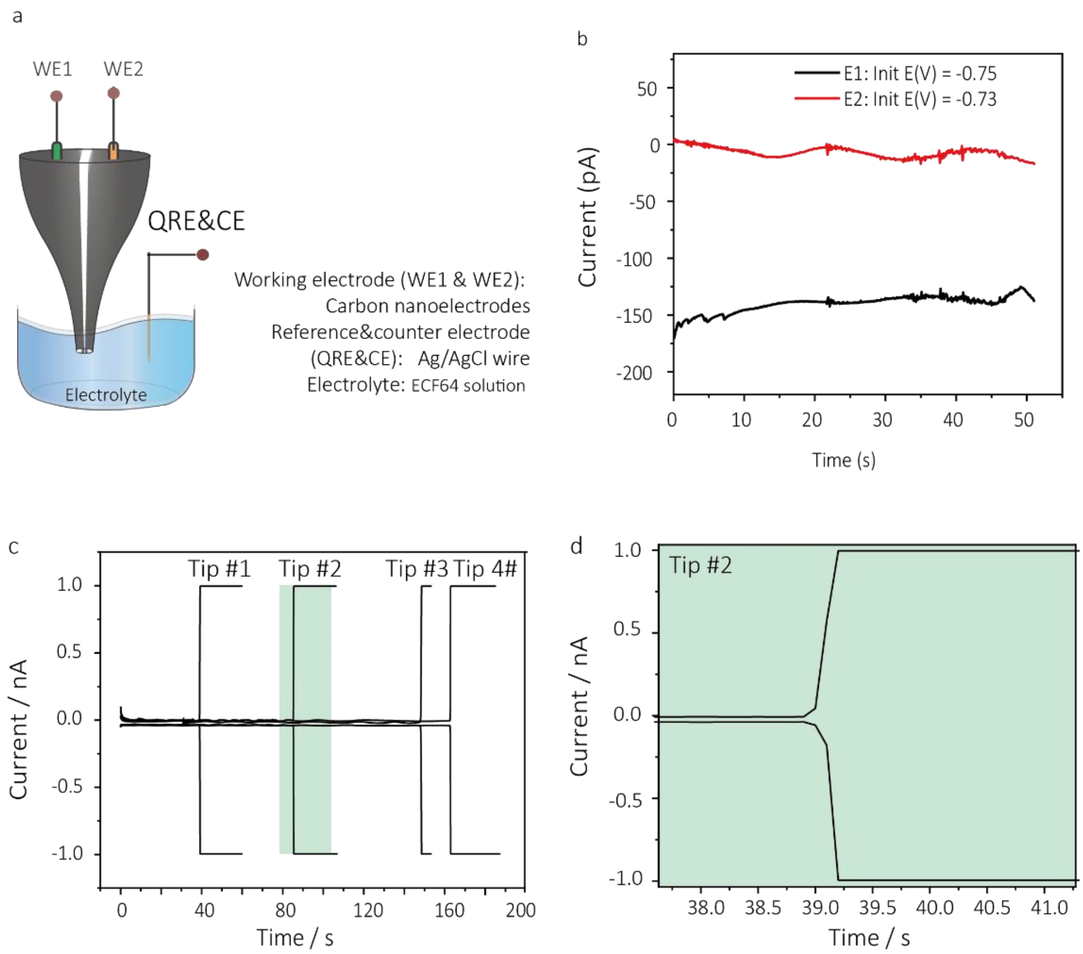

Supplementary Fig. 4| Electrochemical pre-deposition of gold on the carbon nanoelectrodes. (a) Schematic of the bipotentiostatic configuration used for electrodeposition. A diluted ECF64D (Metalor Technologies) plating solution containing 4.4 mM NH_4AuSO_3 in 52 mM $(NH_4)_2SO_3$ was used as the electrolyte and freshly made Ag/AgCl wire (Goodfellow, 0.125 mm OD) was used as the quasi-reference counter electrode (QRE&CE) while the two carbon nanoelectrodes acted as the working electrodes. (b-d) The current-time plot recorded during bipotentiostatic deposition of gold onto carbon electrodes E1 and E2 (deposition time 5 s). Current-time plot recorded during the electrodeposition of gold onto different carbon nanoelectrodes (Tips #1-4). An abruptly increase in both the current was observed during the deposition due to the generation of tunnelling current when the gap distance was close to the tunnelling region leading to electrical contact formation.[5, 7, 8]

Supplementary Note 2 Characterisation of QMT probes

Supplementary 2.1 Electrochemical characterisation of QMT probes

The effective radius of the total gold deposit formed probe tip could be estimated from the steady-state current in bulk solution, as determined by cyclic voltammetry (CV) using hexaammineruthenium(III) chloride ($Ru(NH_3)_6Cl_3$) as a redox mediator.[9] The representative cyclic voltammograms recorded each carbon nanoelectrode showed a characteristic sigmoidal response. Assuming hemispherical diffusion, the steady-state currents (i_{lim}) obtained can be used to calculate the electrode radii using equation 2.1,

$$i_{lim} = 4n_e aFDc \qquad \text{Equation (2.1)}$$

where n_e is the number of electrons transferred, F is the Faraday constant, D = 8.2 x 10^{-6} cm^2 s^{-1} is the diffusion coefficient of $Ru(NH3)_6^{3+}$. c is the concentration of the redox mediator, and a is the electrode radius. The radius of carbon nanoelectrodes calculated from the limiting current was 30 ± 7.8 nm, in good agreement with the initial nanopore diameter of nanopipette tip (SEM imaging seen in the Supplementary Fig. 3b). Whereas, in case of the gold-deposited electrodes a significant increase in the limiting current was observed, indicating the larger surface area introduced by the gold deposition while still small junction electrode with the radius of 339 ± 182 nm. It should be noted that these electrode radius calculations were performed assuming a planar disc-shaped electrode embedded in an infinite insulating plane with an RG ⩾ 10 (RG= rg/a, where rg is the radius of the quartz insulator around the electrode) creating a hemispherical mediator diffusion towards the electrodes.[10, 11] The carbon electrode in the QMT probes described herein are semi-elliptical in shape with recessed electrode geometries with a finite insulating sheath around them (RG ~1.5-2). Hence the electrode radii calculated using the limit currents are only indicative as an approximation of the actual electrode sizes.

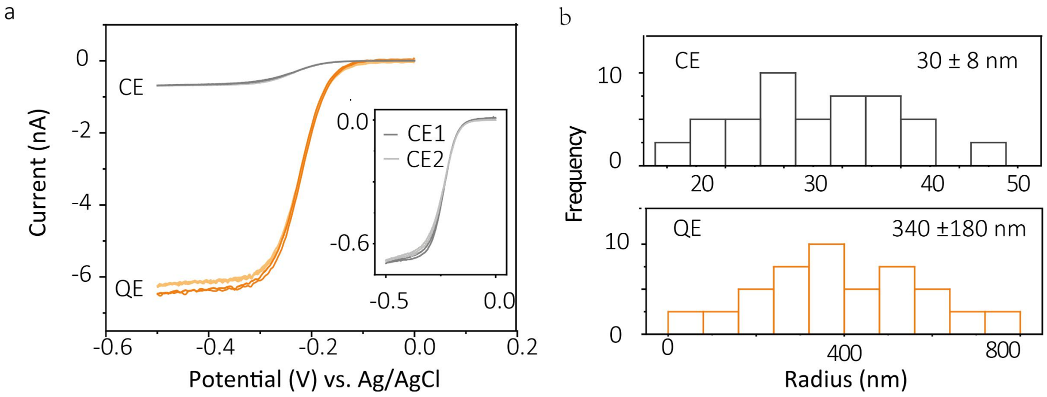

Supplementary Fig. 5| Electrochemical characterisation of carbon nanoelectrodes. (A) Cyclic voltammograms recorded at the two electrodes of a typical QMT probe before (ie, carbon electrode, CE) and after gold deposition (ie, tunnelling electrode,TE). These results were verified further by repeating the experiments at least three times. (B) The distribution of electrode radii calculated from the limiting currents (n= 10 independent measurements for every QMT probes).

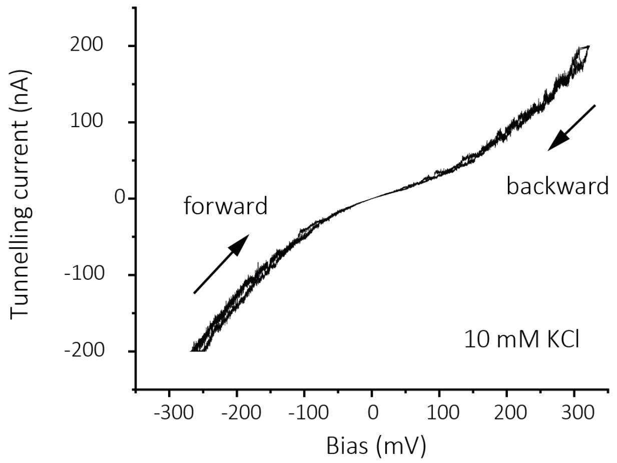

Supplementary Fig. 6| Cyclic voltammogram recorded for a typical tunnelling device in 10 mM KCl solution. Scan rate: 1 V/s. The forward and reverse sweeps were mostly overlapped, indicating the low capacitance effect and good reproducibility.

To examine the influence of Faraday current on the increasing conductance at −0.6V, we carried out Cyclic Voltammetry measurements on individual electrode at a relatively slow scan rate of 1.0 mV/s. The Cyclic Voltammetry demonstrates that the difference of Faraday current at −0.4 V and −0.6 V is lower than 10 pA (Figure S8), indicating no significant Faradaic processes at either electrode (n= 10 QMT devices).[12]

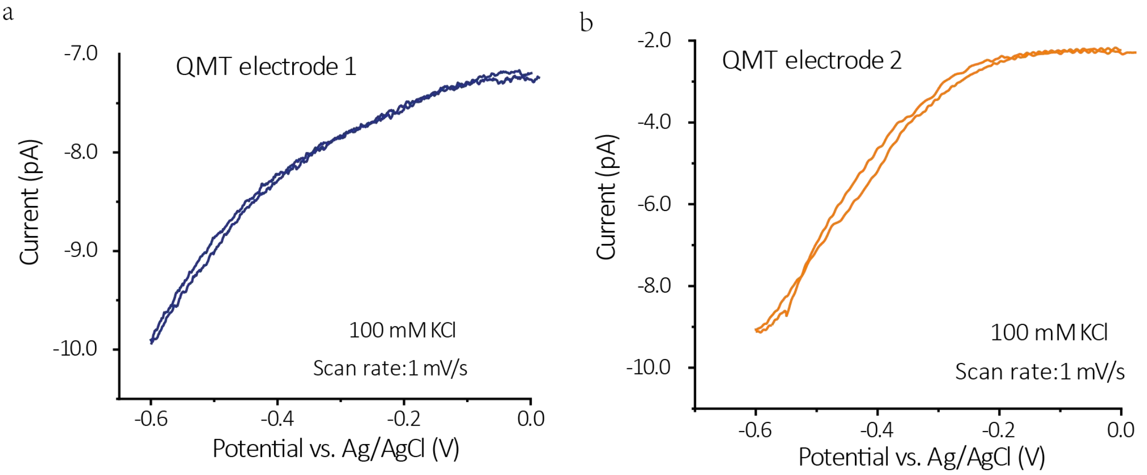

Supplementary Fig. 7| Cyclic voltammogram recorded at the tunnelling nanoelectrodes. Electrolyte: 100 mM KCl; scan rate:1 mV/s; working electrode: one of the tunnelling nanoelectrodes; reference electrode: Ag/AgCl wire; counter electrode: Pt wire.

Supplementary 2.2 Optical and structural characterisation of QMT probes.

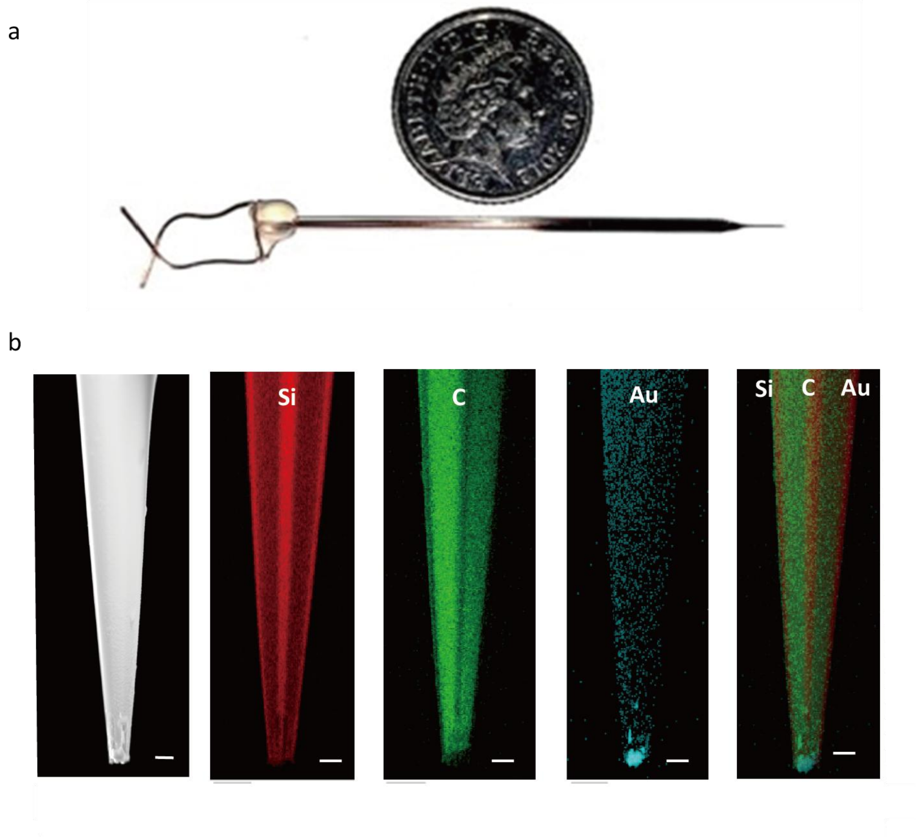

Supplementary Fig. 8| (a) Optical image and (b) STEM-EDX mapping for a QMT device. (b) The STEM-EDX mapping corresponding to Figure 1b showed the presence of silicon, deposited carbon and electrodeposited gold at the QMT probe tip. Scale bar, 50 nm.

Supplementary 2.3 Water reshaping of gold-deposited nanoelectrodes over long time periods

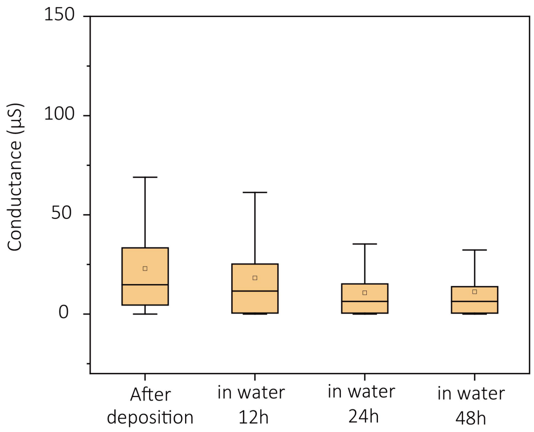

Supplementary Fig. 9| Decrease of tunnelling nanoelectrodes over long time periods. The conductance of each probe was measured at low voltage regime (-50 mV~ 50 mV), where the IV curves were liner. The change in conductance with time indicates the reshaping effect of the water on the tunnelling nanoelectrodes. All error bars represent 1 standard deviation from the mean.

Supplementary Note 3 Functionality characterisation of QMT probes

As the gap width was very small and beyond the resolution of SEM, we could not directly obtain the value. In this work, we performed I-V measurement in a range of different solvents with different barriers hight (air, DI water, n-hexane and dimethyl sulfoxide (DMSO)). The resulting I-V curves could be fitted according to the Simmons model, which is a standard model to describe tunnelling under a potential barrier.[13] In the Simmons model (Equation 1), it is assuming that the work function ϕ of the electrode equals to the barrier height Φ_B, When the bias V is in short-range, eV is smaller than the barrier height Φ_B, effective barrier height $\overline{\phi}$ equals to $\Phi_\mathrm{B} - \mathrm{eV}/2$ and barrier width equals to gap distance d. As the net voltage applied across the junction is increasing, field emission occurs, and the tunnelling current increases exponentially at the same time. In this case, $\overline{\Phi} = \Phi_\mathrm{B}/2$ and $s = d\Phi_\mathrm{B}/\mathrm{eV}$.

$$I = A\left(\frac{\mathrm{e}}{4\pi^2\hbar s^2}\right)\left\{\overline{\phi}\exp\left[-\frac{2(2\mathrm{m_e})^{\frac{1}{2}}}{\hbar}\sqrt{\overline{\phi}}s\right] - \left(\overline{\phi} + \mathrm{eV}\right)\exp\left[-\frac{2(2\mathrm{m_e})^{\frac{1}{2}}}{\hbar}\sqrt{\overline{\phi} + \mathrm{eVs}}\right]\right\} + B \qquad \text{Equation (3.1)}$$

(*d* is the gap distance, *A* the active tunnelling area, and *B* is a parameter that considers any current offset at zero bias due to residual Faradaic processes or minor calibration errors)

The I-V curves for the different QMT device were recorded in air and fit using the Simmons model. IV curves for 144 QMT probes are presented in Supplementary Fig. 10.

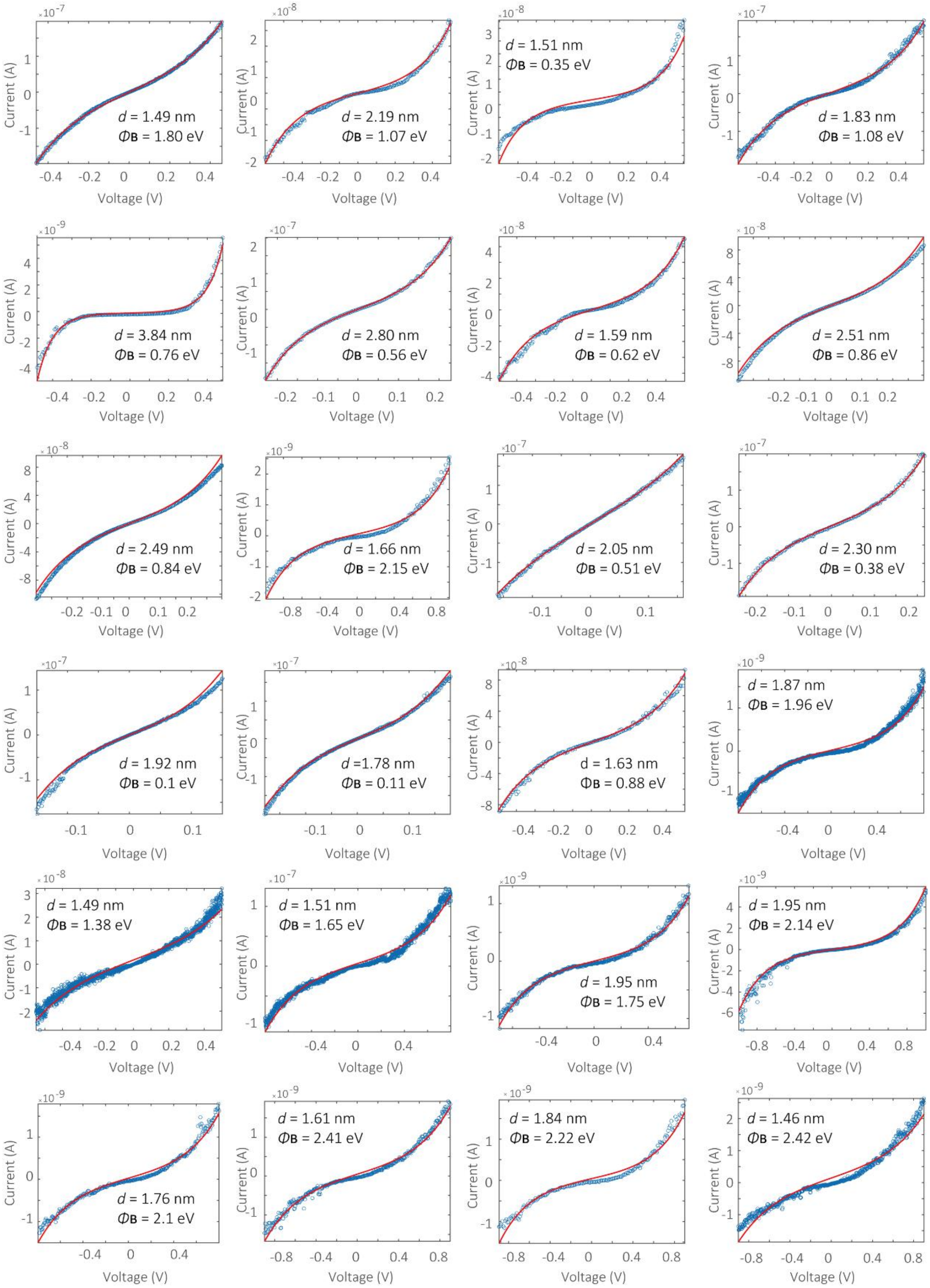

Supplementary Fig. 10a| Tunnelling current measurement of QMT devices (#1-#24) in air at room temperature (297 K) and corresponding fit obtained using the Simmons model.

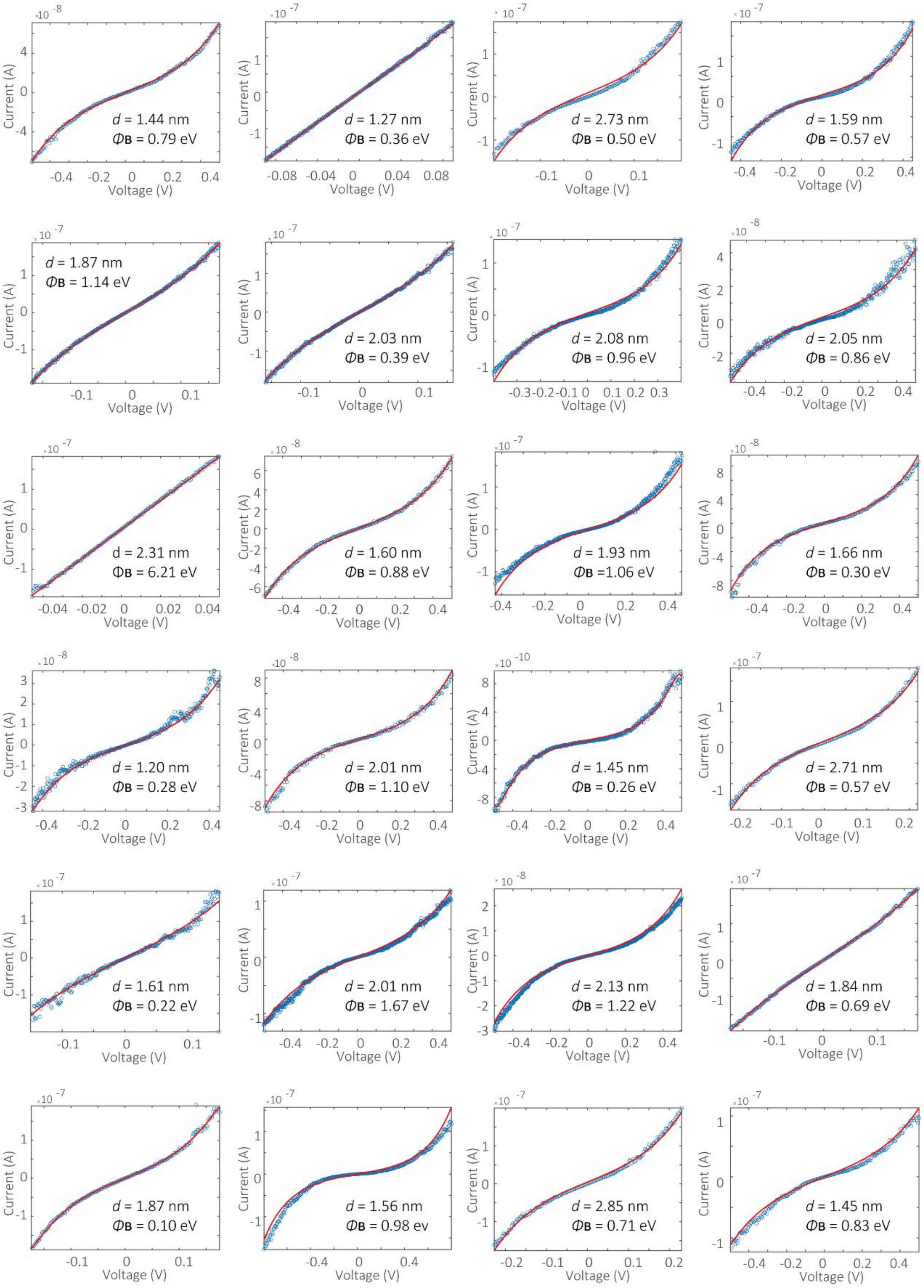

Supplementary Fig. 10b| Tunnelling current measurement of QMT devices (#1-#24) in air at room temperature (297 K) and corresponding fit obtained using the Simmons model.

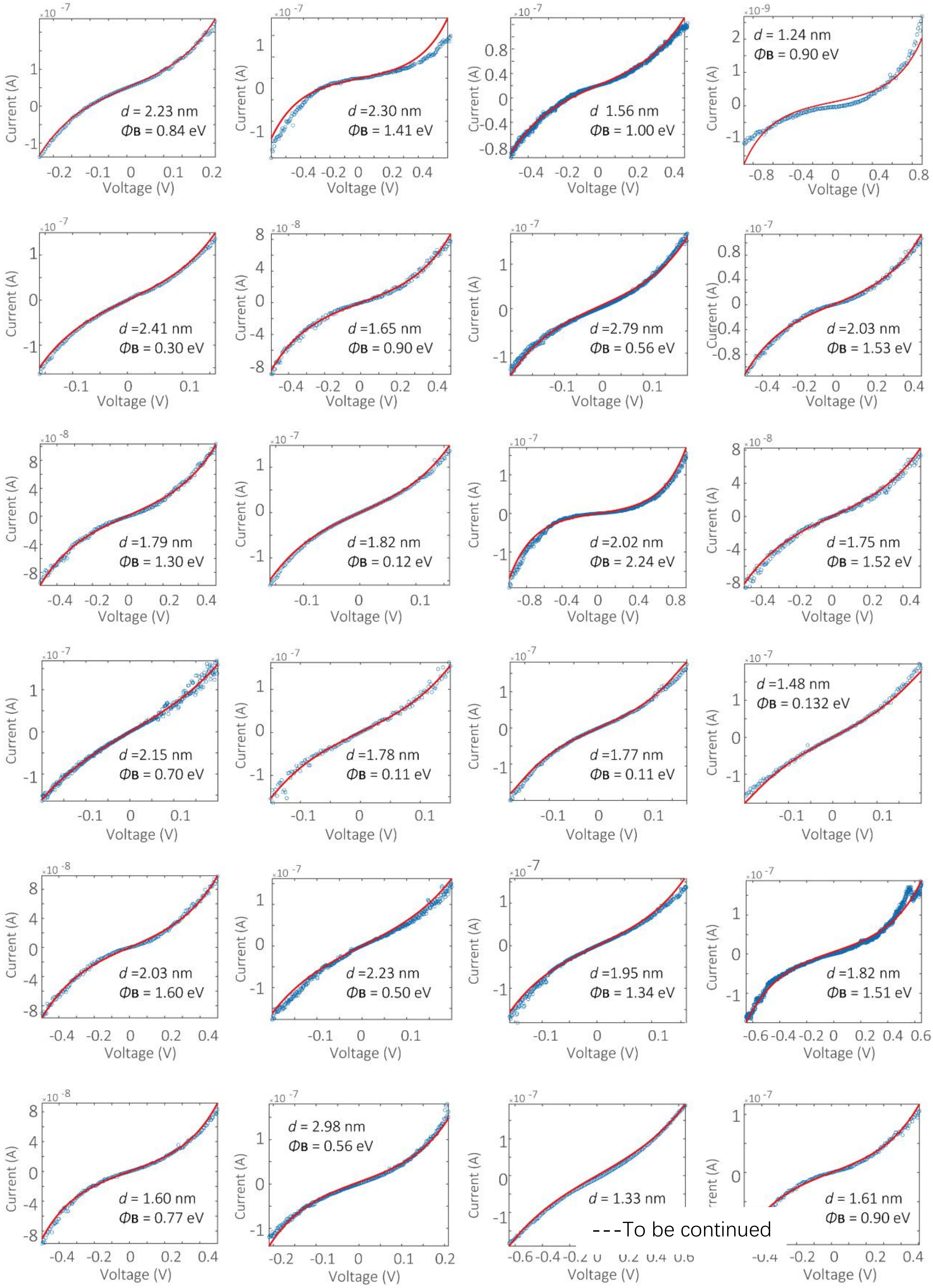

Supplementary Fig. 10c| Tunnelling current measurement of QMT devices (#25-#48) in air at room temperature (297 K) and corresponding fit obtained using the Simmons model.

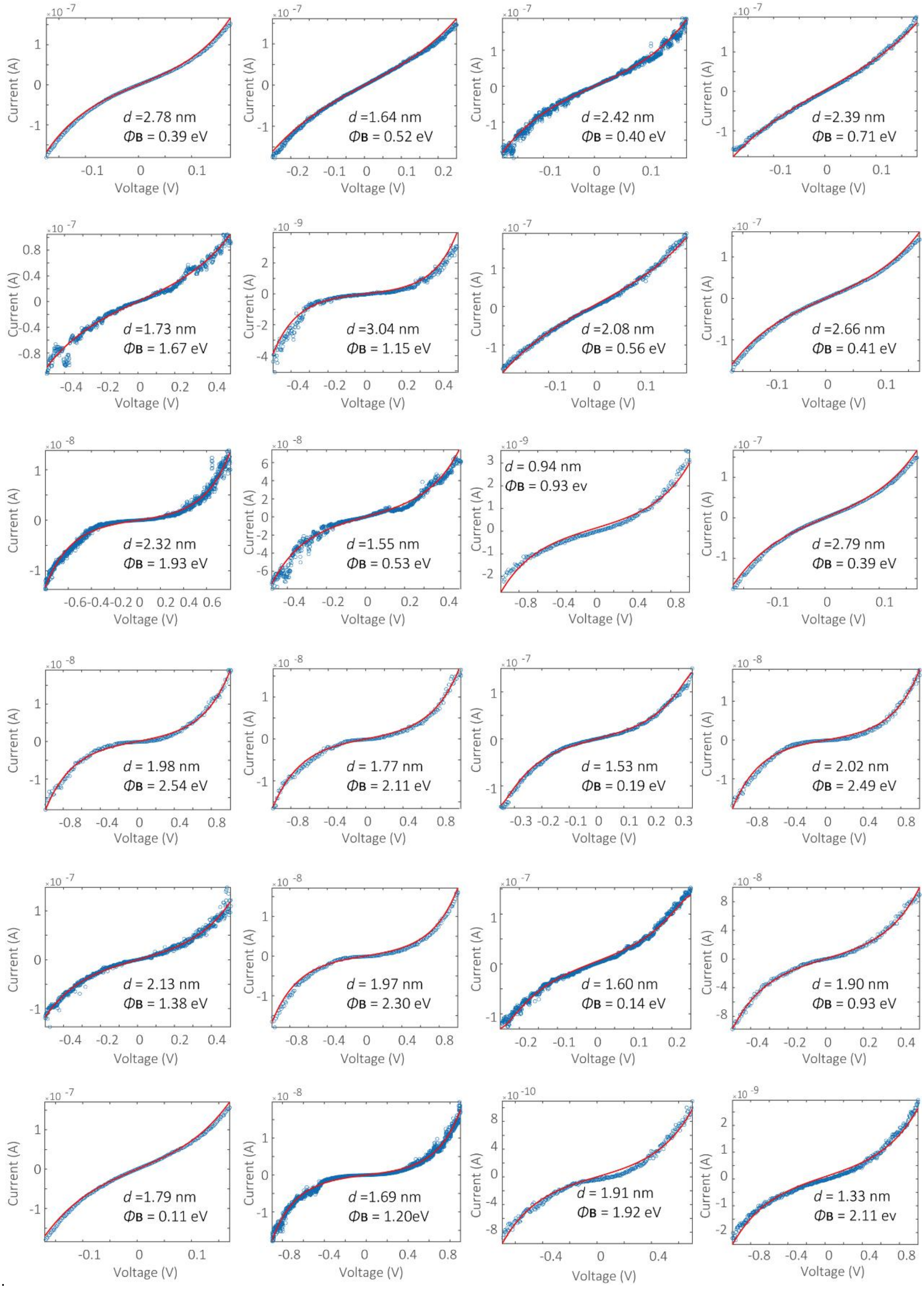

.

Supplementary Fig. 10d| Tunnelling current measurement of QMT devices (#49-#72) in air at room temperature (297 K) and corresponding fit obtained using the Simmons model.

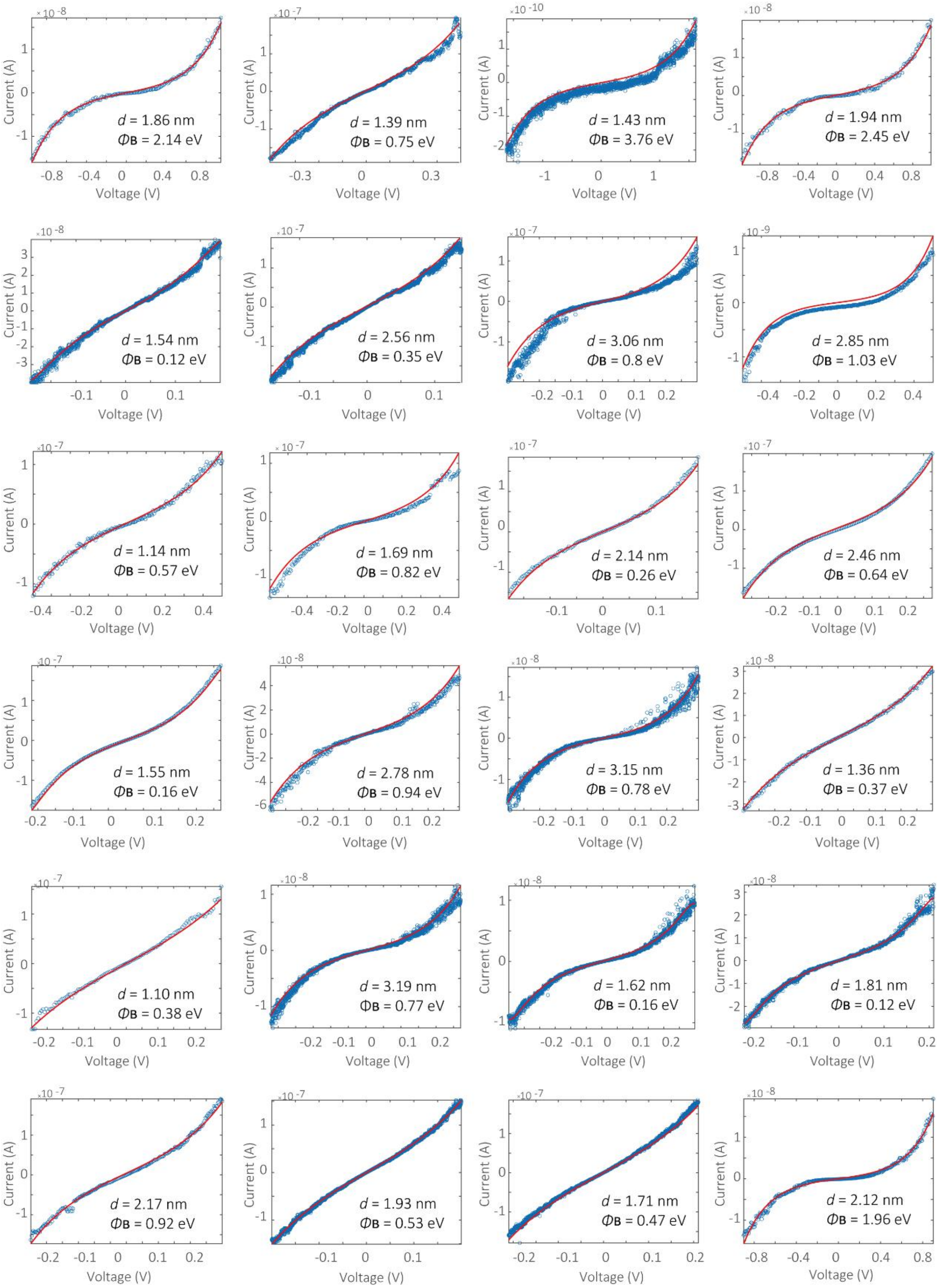

Supplementary Fig. 10e| Tunnelling current measurement of QMT devices (#73-#96) in air at room temperature (297 K) and corresponding fit obtained using the Simmons model.

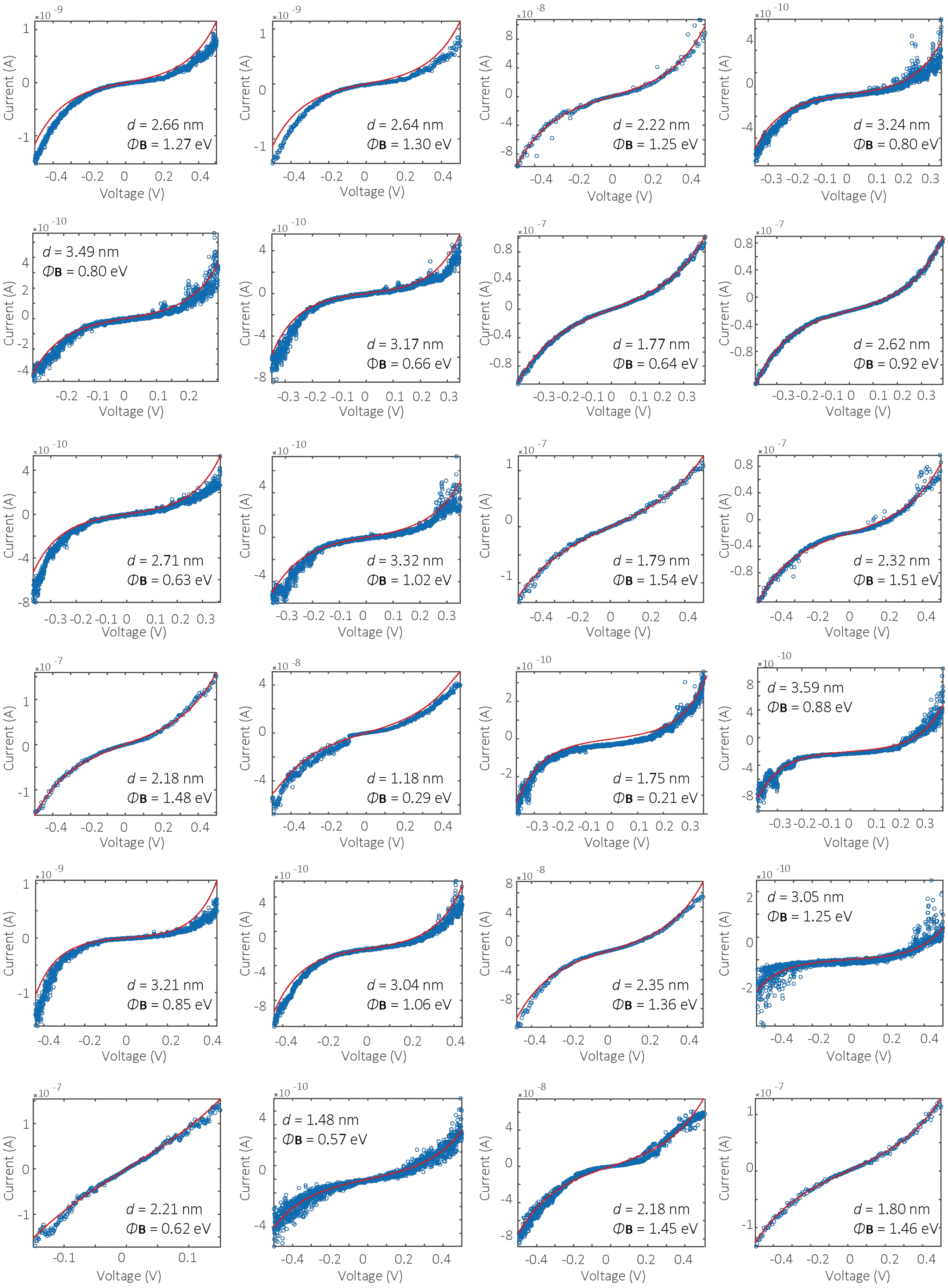

Supplementary Fig. 10f| Tunnelling current measurement of QMT devices (#97-#120) in air at room temperature (297 K) and corresponding fit obtained using the Simmons model.

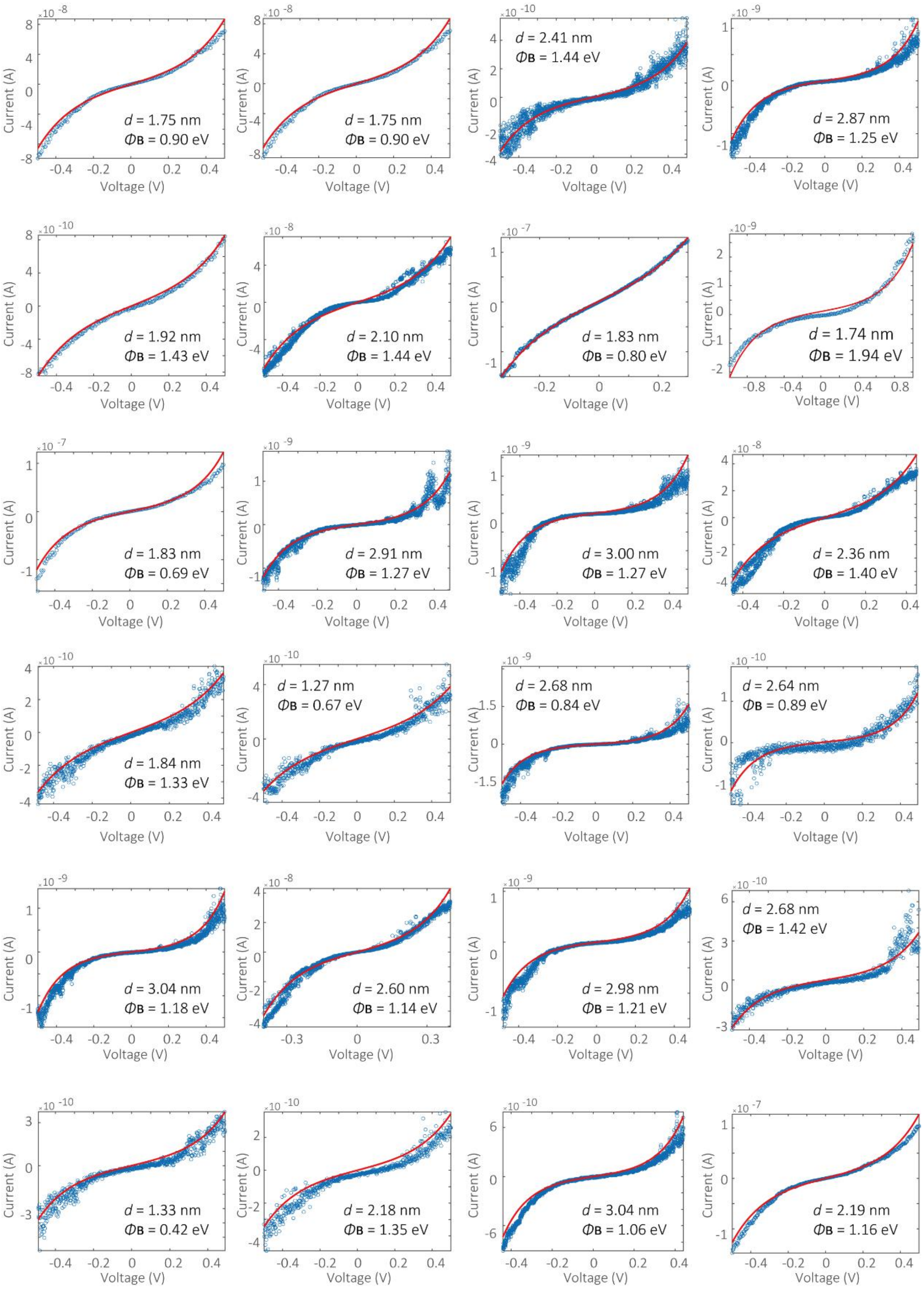

Supplementary Fig. 10g| Tunnelling current measurement of QMT devices (#121-#144) in air at room temperature (297 K) and corresponding fit obtained using the Simmons model.

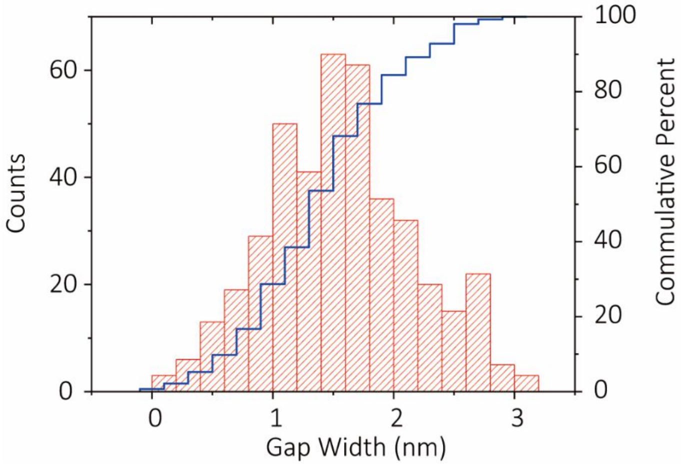

Supplementary Fig. 11| The distribution of tunnelling gap width for QMT devices (N=418). The gap width was obtained by fitting the current-voltage response using the Simmons model.

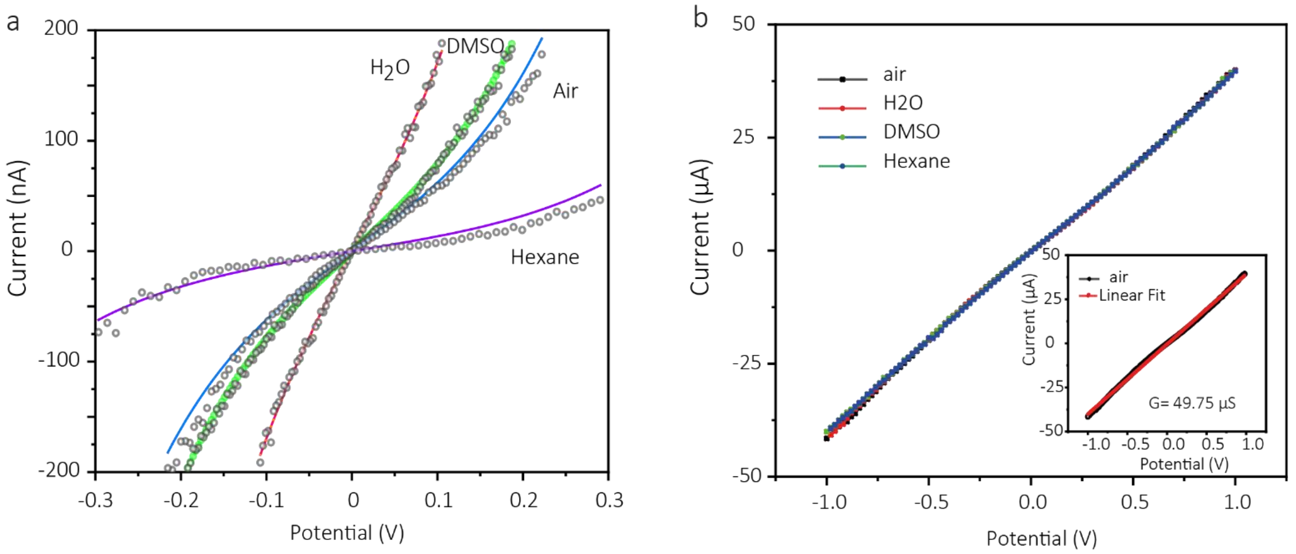

Supplementary Fig. 12| Current-voltage profile recorded for of (a) QMT probe and (b) electrically contacted nanoelectrodes in DI water, dimethyl sulfoxide (DMSO) and hexane. For probes with bridged nanoelectrodes, no solvent dependence was observed.[14, 15]

Supplementary Note 4 QMT detection of different molecules.

Supplementary 4.1 QMT detection of poly-A20 at different concentrations.

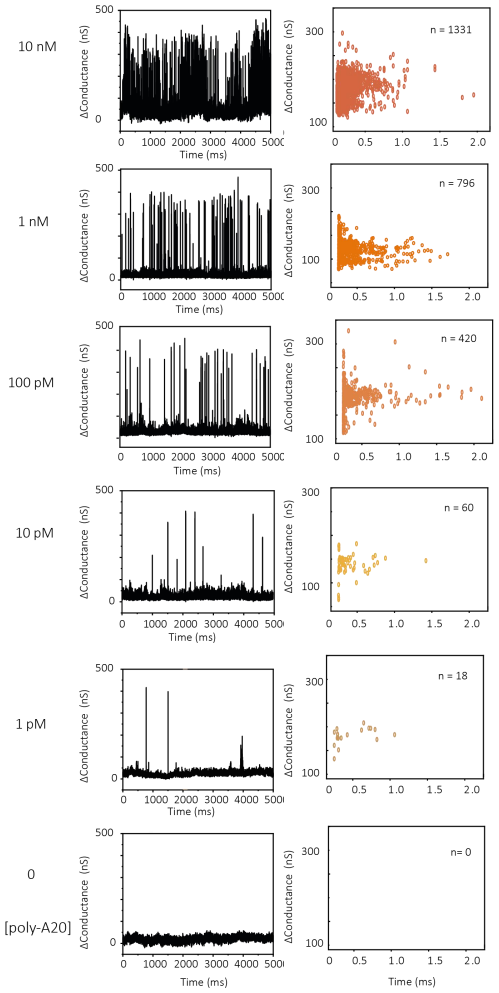

Supplementary Fig. 13| Tunneling detection of poly-A20 using QMT probes and varying concentrations. Experiments were performed in 1 mM PBS (pH 7.4) containing varying concentrations of poly-A20 (1 pM to 10 nM). In all experiments, a bias of 50 mV was used. Before each measurement, the device was rinsed with DI water to confirm the clean background. The left panels show conductance time traces whilst the right panels are scatter plots corresponding to individual events.

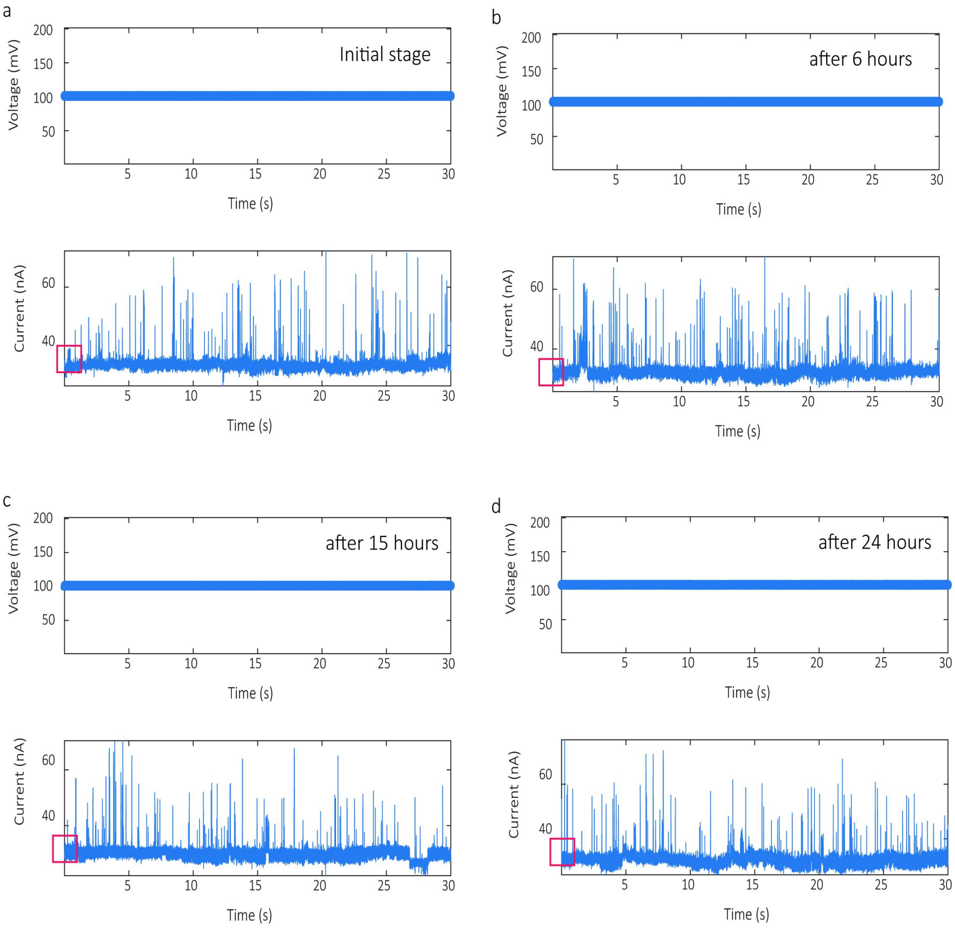

Supplementary Fig. 14| Long-term stability of the QMT probes. Measurements were performed using 10 nM poly-A20 over a 24 hr period.

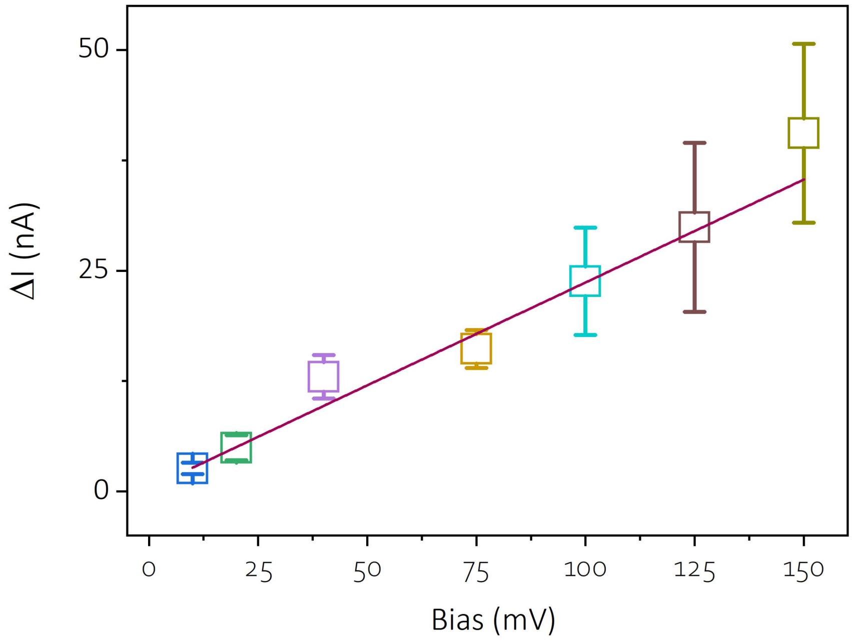

Supplementary Fig. 15| Plots of peak current versus bias voltage for data presented in Figure 3e. All error bars represent 1 standard deviation from the mean.

Supplementary 4.2 QMT detection of poy-A20 using tunnelling probes with different gap width.

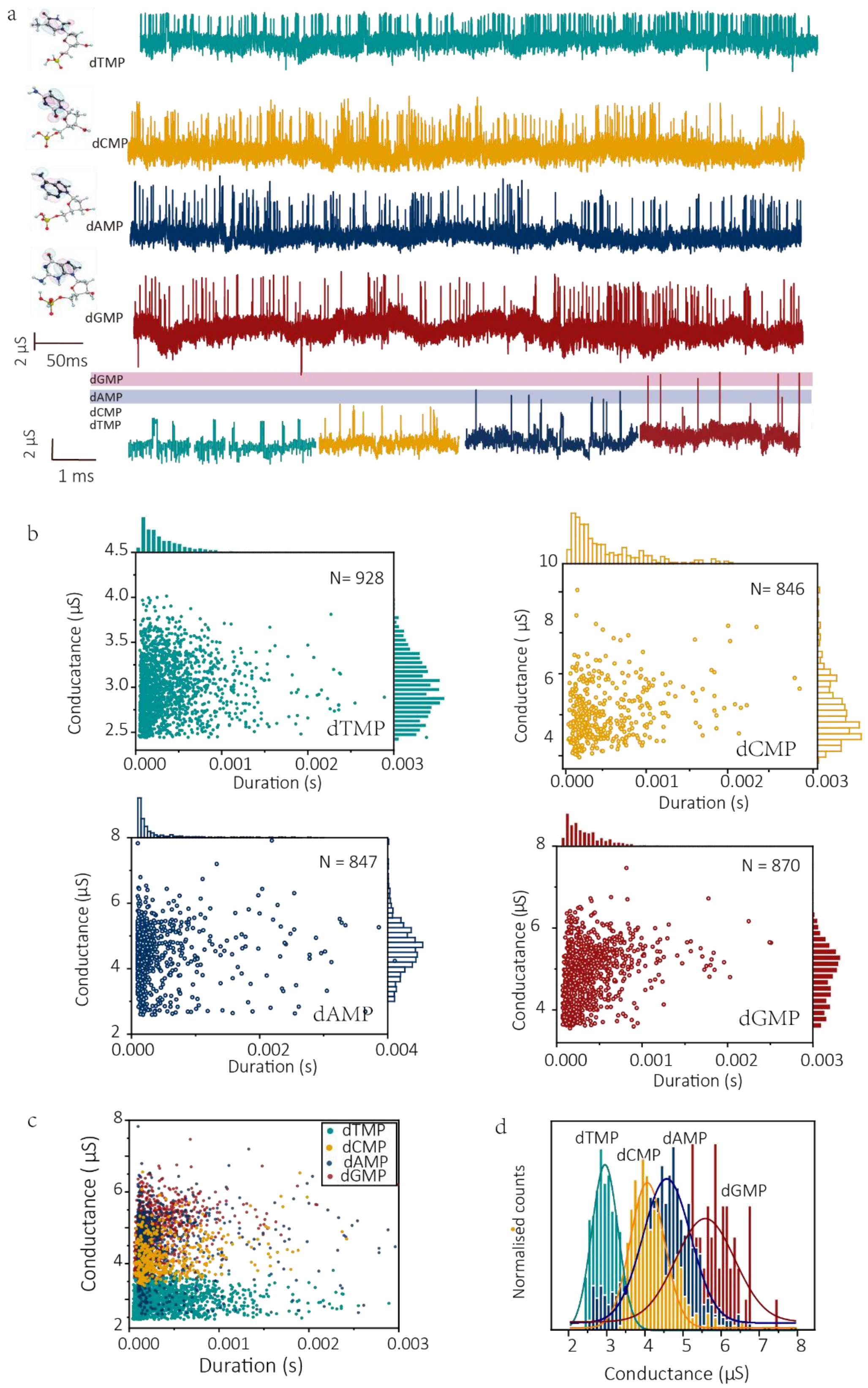

Supplementary Fig. 16| Tunnelling detection of single nucleotides using the QMT probe with 0.7 nm gap. Data were acquired in 1 mM PBS (pH 7.4) containing 100 pM nucleotides, at applied bias of 10 mV. (a) Conductance-time traces recorded for each nucleotide: dTMP (green), dCMP (orange), dAMP (blue) and dGMP (red) for two different durations (0.5 and 0.1 s). (b, c) Scatter plots of the transients for each mononucleotide, showing distinct conductance for dAMP, dGMP, dCMP and dTMP respectively. (d) Normalised histogram of conductance change for each nucleotide. Mean values are shown in Supplementary Table S2.

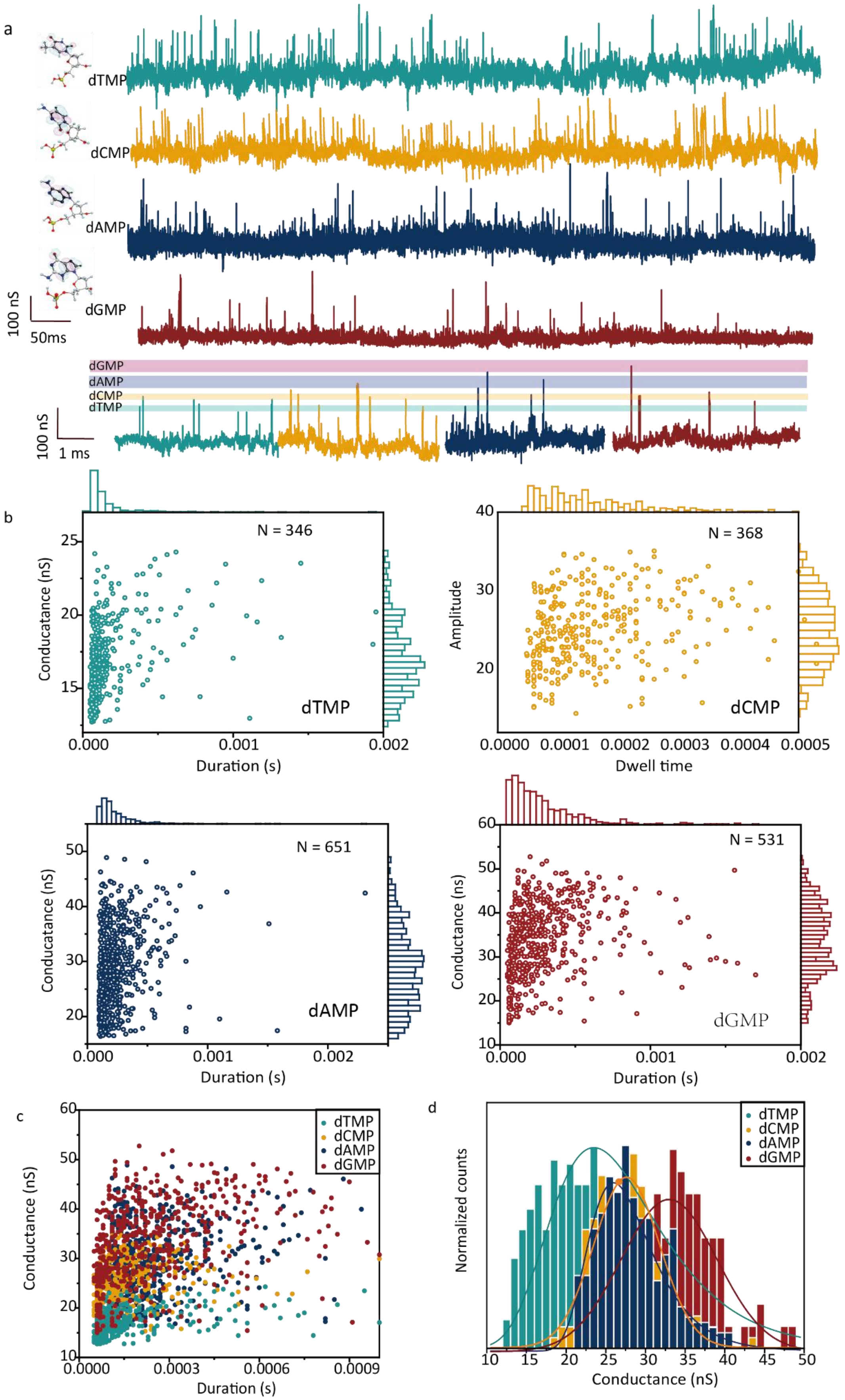

Supplementary Fig. 17| Tunnelling detection of single nucleotides using the QMT probe with 1.9 nm gap. Data were acquired in 1 mM PBS (pH 7.4) containing 100 pM nucleotides, at 200 mV. (a) Conductance-time traces recorded for each nucleotide: dTMP (green), dCMP (orange), dAMP (blue) and dGMP (red) for two different durations (0.5 and 0.1 s). (b, c) Scatter plots of the transients for each mononucleotide, showing distinct conductance for dAMP, dGMP, dCMP and dTMP respectively. (d) Normalised histogram of the conductance change for each nucleotide. Mean values are shown in Supplementary Table S2.

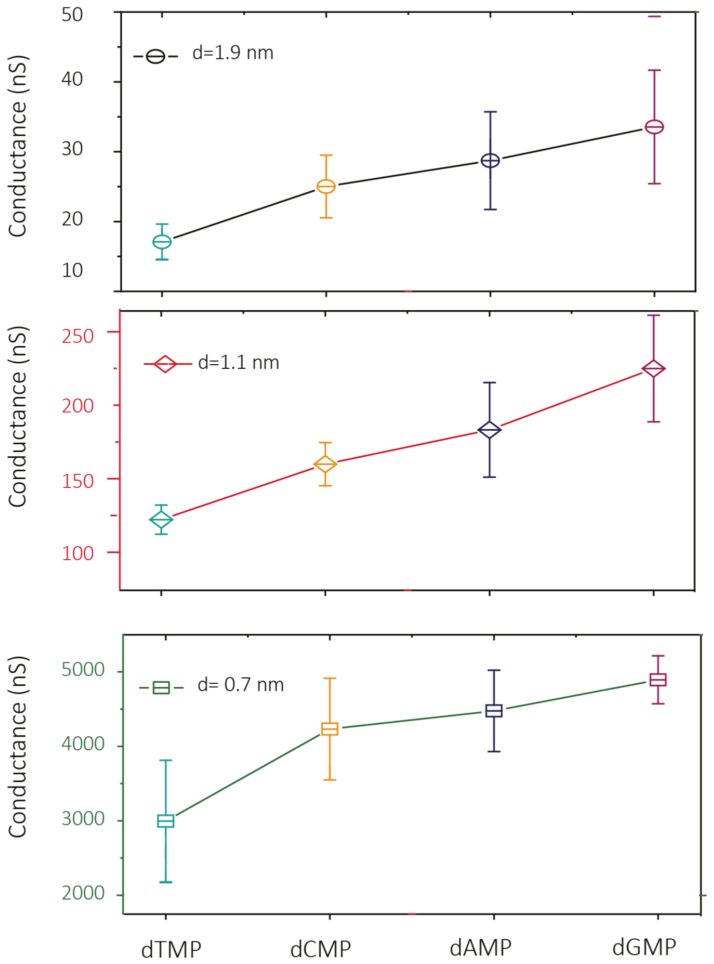

Supplementary Fig. 18| Comparison of the conductance change for each nucleotide using QMT probes with gaps widths with different size regimes. Nucleotides measured with QMTs with different gap widths exhibited similar conductance trends. All error bars represent 1 standard deviation from the mean.

Table S2. Conductance and dwell time for each nucleotide obtained from different QMT probes with gaps widths with different size regimes.

	Junction d = 1.9 nm		Junction d = 1.1 nm		Junction d = 0.7 nm	
	ΔG (ns)	Δt (ms)	ΔG (ns)	Δt (ms)	ΔG (ns)	Δt (ms)
dTMP	17 ± 3	0.19	120 ± 10	0.25	3000 ± 300	0.34
dCMP	25 ± 4	0.17	161 ± 5	0.24	4200 ± 500	0.40
dAMP	29 ± 7	0.24	180 ± 30	0.15	4500 ± 800	0.50
dGMP	34 ±8	0.31	240 ± 40	0.23	5300 ± 400	0.46

.

.

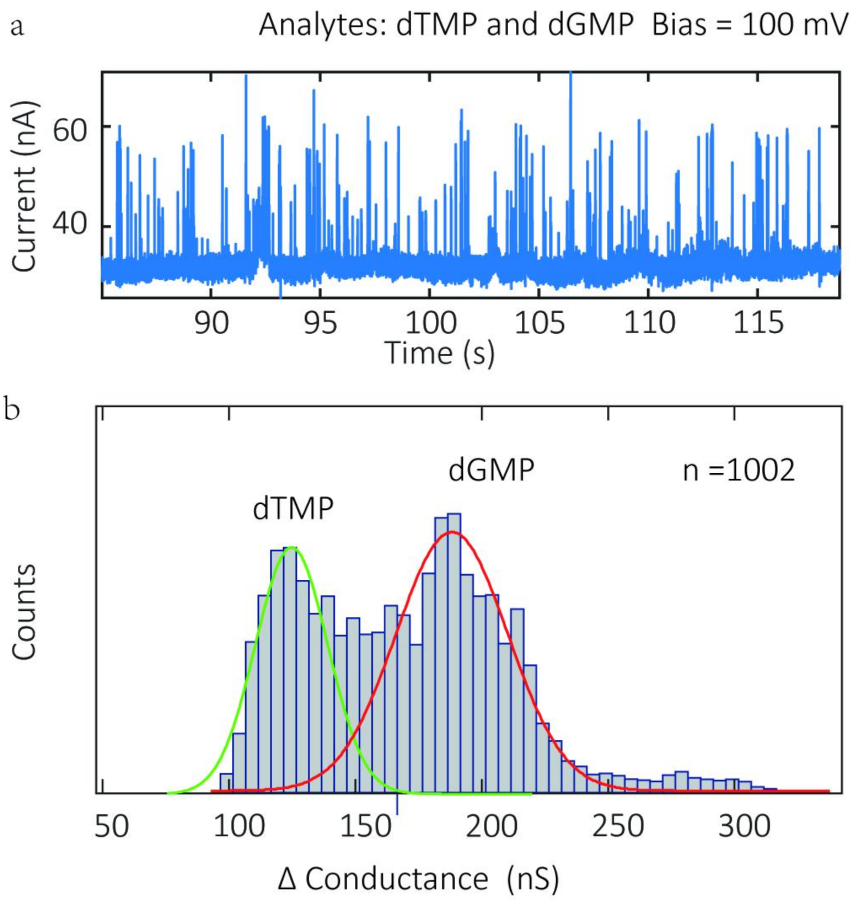

Supplementary Fig. 19| (a) Current-time trace and transients conductance histogram obtained for a solution containing equimolar amounts (100 pM) of dGMP and dTMP. Solid curves are Gaussian fits, revealing two peaks for the dTMP and dGMP

Supplementary Note 5 Dielectrophoretic - tunnelling detection using QMT probes

Supplementary 5.1 Stability evaluation of QMT probes under DEP trapping

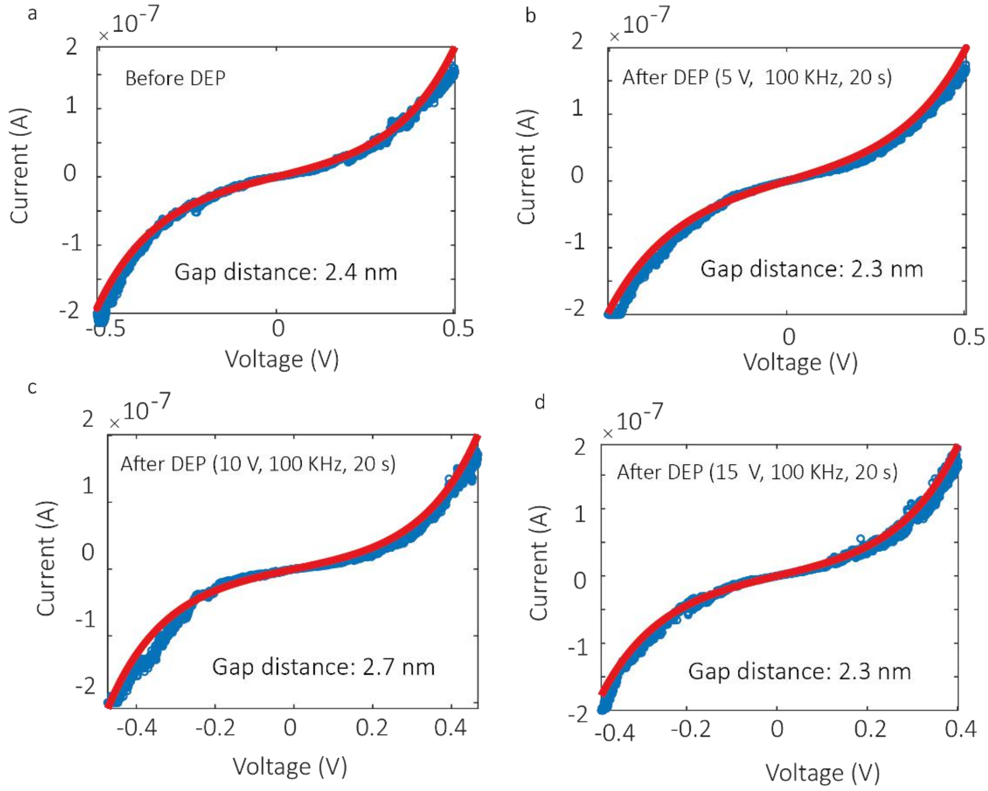

Supplementary Fig. 20| IV stability measurements for QMT probe before and after DEP trapping. IV curves were measured for the QMT probe a) before and after DEP trapping at b) $5V_{p\text{-}p}$ c) $10V_{p\text{-}p}$ and d) $15V_{p\text{-}p.}$ Relatively small changes in the current and fitted gap distance demonstrated indicated high stability of the tunnelling nanoelectrodes.

Supplementary 5.2 Fluorescence imaging and characterisation of QMT probes with DEP trapping

All fluorescence images and videos were acquired using a custom-built optical microscope.[16] In brief, a continuous-wave solid-state laser (λ_{EX} = 488 nm, Sapphire 488LP, Coherent) was used to illuminate the sample. The emitted light was imaged using an emCCD camera (iXon Ultra 897, Andor Technologies). A controller unit (ProScan II, Prior Scientific) was used to precisely control the motorised stage (H1117, Prior Scientific) and the z-axis focus motor, ensuring accurate sample position. A custom dichroic filter (Chroma Technology) was used to isolate the laser excitation source from the emitted fluorescence light. The QMP probe was mounted on a single-axis miniature translational stage (DT12, ThorLabs) and used to manually approach the QMT probe to within the working distance of the objective. The translational stage was placed on top of a motorised stage (Prior Scientific) consisting of a universal sample holder (H473XR, Prior Scientific).

To perform DEP trapping, the DEP field was generated at the QMT tip by applying an AC voltage between the electrodes using a standard function generator (ATG2000, TTi UK). After the DEP was switched off, tunnelling currents were measured using a patch-clamp (MultiClamp 700B, Molecular Devices, USA). Labelled DNA samples for imaging were prepared by incubating 250 pM DNA solution in 10 mM Tris 1 mM EDTA with YOYO-1 (Molecular Probes) at a ratio of 1 YOYO-1 molecule per five base pairs. Streptavidin was purchased prelabelled with Alexa-488 fluorophore (Sigma-Aldrich) and diluted as needed.

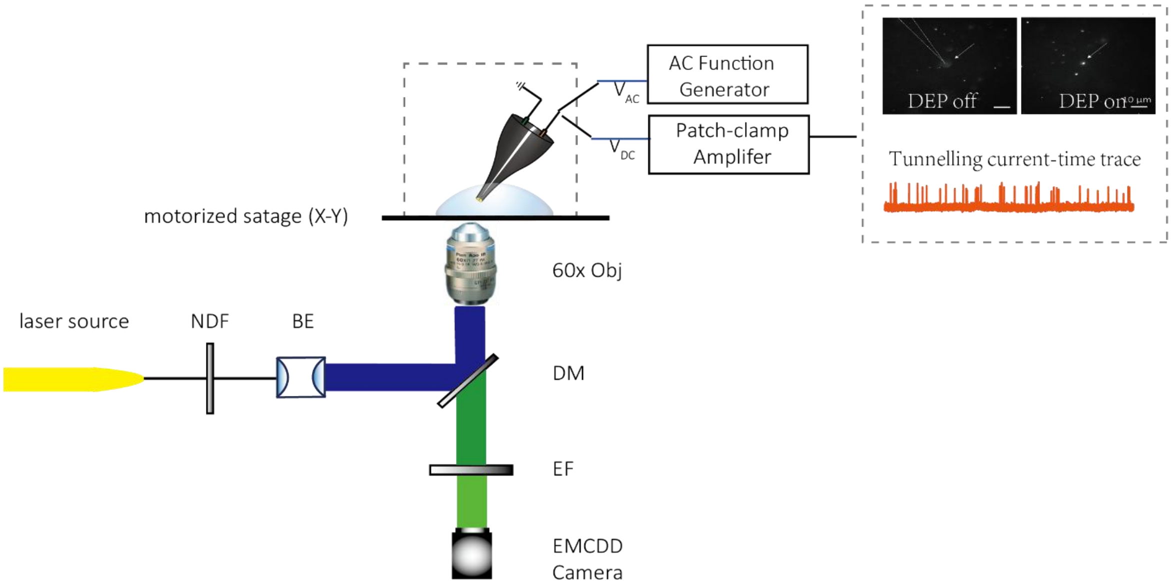

Supplementary Fig. 21| Schematic of the setup used for fluorescence and tunnelling monitoring of QMT detection under DEP trapping.

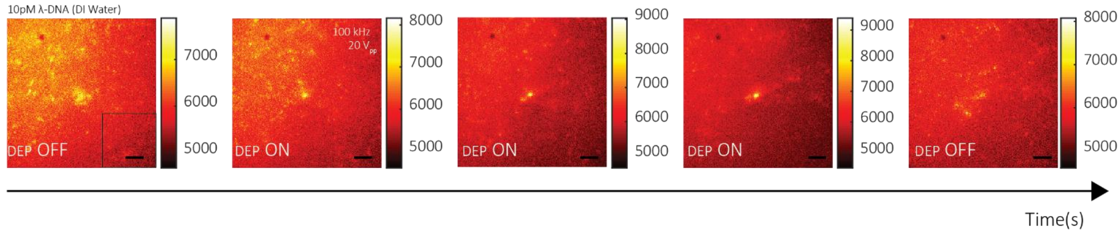

Supplementary Fig. 22| Fluorescence images recorded at different time intervals during the DEP trapping of lambda-DNA. The YOYO-1 labelled lambda-DNA (10 pM in DI water) was used. f_{AC}: 100 kHz; V_{pp}: 10 V; Scale bars: 4 µm.

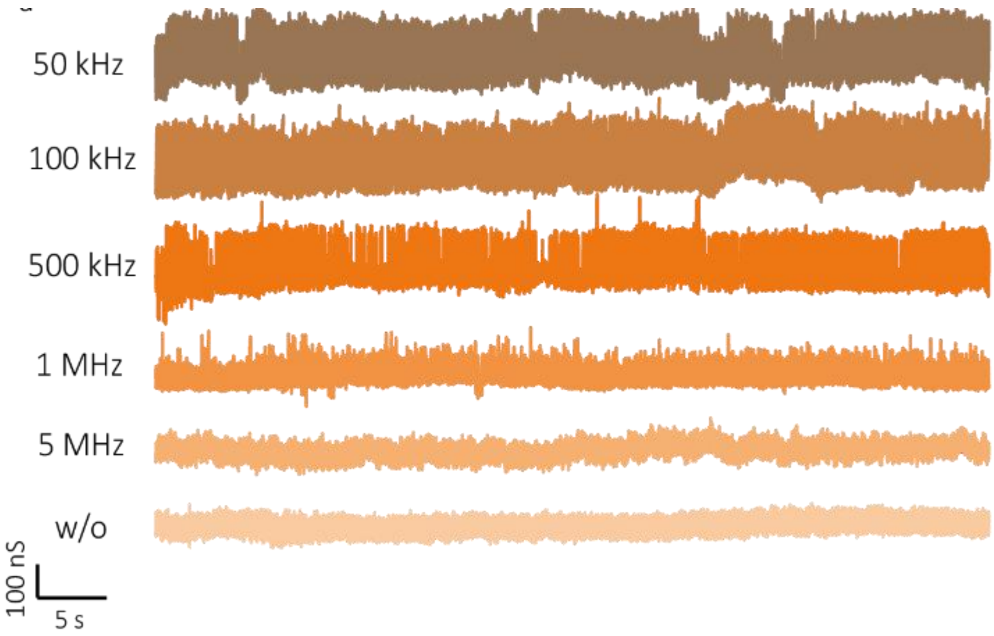

Supplementary Fig. 23| Typical conductance -time profiles of QMT detection of poly-A20 under different AC frequency. All DEP data were obtained at trapping condition V_{pp} =10 V and t = 10 s. The concentration of poly-A20 was 1 fM in 1 mM PBS (pH 7.4), and measurement was performed at 50 mV applied bias.

Supplementary References

1. Cadinu P, Campolo G, Pud S, Yang W, Edel JB, Dekker C*, et al.* Double Barrel Nanopores as a New Tool for Controlling Single-Molecule Transport. *Nano Letters* 2018, **18**(4)**:** 2738-2745.

2. Cadinu P, Paulose Nadappuram B, Lee DJ, Sze JYY, Campolo G, Zhang Y*, et al.* Single Molecule Trapping and Sensing Using Dual Nanopores Separated by a Zeptoliter Nanobridge. *Nano Lett* 2017, **17**(10)**:** 6376-6384.

3. Nadappuram BP, Cadinu P, Barik A, Ainscough AJ, Devine MJ, Kang M*, et al.* Nanoscale tweezers for single-cell biopsies. *Nat Nanotechnol* 2019, **14**(1)**:** 80-88.

4. Xue L, Cadinu P, Paulose Nadappuram B, Kang M, Ma Y, Korchev Y*, et al.* Gated Single-Molecule Transport in Double-Barreled Nanopores. *ACS Appl Mater Interfaces* 2018, **10**(44)**:** 38621-38629.

5. Yu LH, Natelson D. Zero-bias anomalies in electrochemically fabricated nanojunctions. 2003, **82**(14)**:** 2332-2334.

6. Chen S, Kucernak A. Fabrication of carbon microelectrodes with an effective radius of 1 nm. *Electrochemistry Communications* 2002, **4**(1)**:** 6.

7. Deshmukh MM, Prieto AL, Gu Q, Park H. Fabrication of Asymmetric Electrode Pairs with Nanometer Separation Made of Two Distinct Metals. *Nano Letters* 2003, **3**(10)**:** 1383-1385.

8. Morpurgo AF, Marcus CM, Robinson DB. Controlled fabrication of metallic electrodes with atomic separation. 1999, **74**(14)**:** 2084-2086.

9. Schuster R. Book Review: Scanning Electrochemical Microscopy. Edited by Allen J. Bard and Michael V. Mirkin. 2002, **41**(4)**:** 657-658.

10. Shao YH, Mirkin MV. Probing ion transfer at the liquid/liquid interface by scanning electrochemical microscopy (SECM). *Journal of Physical Chemistry B* 1998, **102**(49)**:** 9915-9921.

11. Bard AJ, Mirkin MV. *Scanning Electrochemical Microscopy*, 2nd edn. CRC Press, 2012.

12. Bai J, Daaoub A, Sangtarash S, Li X, Tang Y, Zou Q*, et al.* Anti-resonance features of destructive quantum interference in single-molecule thiophene junctions achieved by electrochemical gating. *Nat Mater* 2019, **18**(4)**:** 364-369.

13. Simmons JG. Generalised Formula for the Electric Tunnel Effect between Similar Electrodes Separated by a Thin Insulating Film. *Journal of Applied Physics* 1963, **34**(6)**:** 1793-1803.

14. Ivanov AP, Freedman KJ, Kim MJ, Albrecht T, Edel JB. High precision fabrication and positioning of nanoelectrodes in a nanopore. *ACS Nano* 2014, **8**(2)**:** 1940-1948.

15. Ivanov AP, Instuli E, McGilvery CM, Baldwin G, McComb DW, Albrecht T*, et al.* DNA tunneling detector embedded in a nanopore. *Nano Lett* 2011, **11**(1)**:** 279-285.

16. Cadinu P, Kang M, Nadappuram BP, Ivanov AP, Edel JB. Individually Addressable Multi-nanopores for Single-Molecule Targeted Operations. *Nano Letters* 2020, **20**(3)**:** 2012-2019.
