# Supplementary material for: Combined quantum tunnelling and dielectrophoretic trapping for molecular analysis at ultra-low analyte concentrations
Source: Nat Commun. 2021 Feb 10;12:913. doi: 10.1038/s41467-021-21101-x (PMC7876030; doi:10.1038/s41467-021-21101-x)
Supplement: Supplementary file 2 — Description of Additional Supplementary Files [file 41467_2021_21101_MOESM2_ESM.pdf]

Description of Additional Supplementary Files

File Name: Supplementary Movie **1**
Description: Tunnelling detection of poly-A20 using a QMT probe. Video showing tunnelling current-time trace for 100 pM poly-A20 DNA in 1 mM phosphate buffer (PBS, pH = 7.4) using a QMT probe at a potential of 80 mV. The corresponding current increase represents a single DNA molecule residing between the tunnelling junction. Upon a molecule coming into close proximity, interacting, or bridging the gap, current fluctuations in the form of characteristic spike-like transients were observed.

File Name: Supplementary Movie **2**
Description: DEP trapping of lamda DNA using a QMT probe. Video showing the pick and release of single lamdaDNA molecule using the QMT probe. The DNA molecule was captured at the QMT tip by turning on the a.c. field (DEP on). Turning off the DEP resulted in the release of the captured molecule from the QMT tip into the solution
